# Supplementary material for: Radon exposure and COVID-19 mortality in pre-vaccination period: What links might exist?
Source: PLoS One. 2025 Dec 5;20(12):e0337320. doi: 10.1371/journal.pone.0337320 (PMC12680143; doi:10.1371/journal.pone.0337320)
Supplement: S1 Table — This table provides a detailed breakdown of the statistical analysis, exploring the relationship between population density, radon exposure, and mortality rates across various regions in France using a matching pair differential. (PDF) [file pone.0337320.s001.pdf]

Dep Code	Dep Name	Dens (km^2)	pop	Radon Avg (Bq/m^3)	Hosp/10^5	Dep Code	Dep Name	Dens (km^2)	pop	Radon Avg (Bq/m^3)	Hosp/10^5	Δ Dens (km^2)	Δ Radon (Bq/m^3)	Δ Hosp (/10^5)	sign cons.
5	Hautes-Alpes	25,4	141284	144	14,16	15	Cantal	25,5	145143	161	6,20	0,1	17	-8,0	-1
55	Meuse	30,4	187187	62	55,03	32	Gers	30,5	191091	66	12,04	0,1	4	-43,0	-1
32	Gers	30,5	191091	66	12,04	58	Nièvre	30,7	207182	115	13,51	0,2	49	1,5	1
58	Nièvre	30,7	207182	115	13,51	55	Meuse	30,4	187187	62	55,03	0,3	-53	41,5	-1
36	Indre	32,9	222232	102	36,90	46	Lot	33,2	173828	88	12,66	0,3	-14	-24,2	1
43	Haute-Loire	45,7	227283	157	7,92	24	Dordogne	45,8	413606	79	3,38	0,1	-78	-4,5	1
24	Dordogne	45,8	413606	79	3,38	89	Yonne	45,9	338291	68	28,08	0,1	-11	24,7	-1
89	Yonne	45,9	338291	68	28,08	3	Allier	46,2	337988	145	12,13	0,3	77	-16,0	-1
3	Allier	46,2	337988	145	12,13	24	Dordogne	45,8	413606	79	3,38	0,4	-66	-8,7	1
43	Haute-Loire	45,7	227283	157	7,92	89	Yonne	45,9	338291	68	28,08	0,2	-89	20,2	-1
65	Hautes-Pyrénées	51	228530	108	11,81	10	Aube	51,4	310020	35	44,19	0,4	-73	32,4	-1
39	Jura	52,1	260188	92	23,06	41	Loir-et-Cher	52,5	331915	70	19,58	0,4	-22	-3,5	1
41	Loir-et-Cher	52,5	331915	70	19,58	8	Ardennes	52,7	273579	95	21,93	0,2	25	2,3	1
7	Ardèche	58,8	325712	134	35,61	16	Charente	59,3	352335	90	3,69	0,5	-44	-31,9	1
16	Charente	59,3	352335	90	3,69	53	Mayenne	59,5	307445	96	15,61	0,2	6	11,9	1
53	Mayenne	59,5	307445	96	15,61	11	Aude	59,9	370260	86	15,93	0,4	-10	0,3	-1
11	Aude	59,9	370260	86	15,93	7	Ardèche	58,8	325712	134	35,61	0,5	44	31,9	1
47	Lot-et-Garonne	62,1	332842	69	3,61	86	Vienne	62,4	436876	91	9,16	0,3	22	5,6	1
86	Vienne	62,4	436876	91	9,16	79	Deux-Sèvres	62,5	374351	103	5,88	0,1	12	-3,3	-1
79	Deux-Sèvres	62,5	374351	103	5,88	88	Vosges	62,9	367673	135	72,62	0,4	32	66,7	1
88	Vosges	62,9	367673	135	72,62	86	Vienne	62,4	436876	91	9,16	0,5	-44	-63,5	1
47	Lot-et-Garonne	62,1	332842	69	3,61	79	Deux-Sèvres	62,5	374351	103	5,88	0,4	34	2,3	1
80	Somme	92,8	572443	42	40,70	17	Charente-Maritime	93,6	644303	45	7,92	0,8	3	-32,8	-1
37	Indre-et-Loire	98,9	606511	60	15,33	45	Loiret	99,5	678105	55	15,34	0,6	-5	0,0	-1
45	Loiret	99,5	678105	55	15,34	27	Eure	99,8	601843	45	14,12	0,3	-10	-1,2	1
27	Eure	99,8	601843	45	14,12	85	Vendée	99,8	675247	83	6,37	0	38	-7,8	-1
85	Vendée	99,8	675247	83	6,37	45	Loiret	99,5	678105	55	15,34	0,3	-28	9,0	-1
85	Vendée	99,8	675247	83	6,37	37	Indre-et-Loire	98,9	606511	60	15,33	0,9	-23	9,0	-1
37	Indre-et-Loire	98,9	606511	60	15,33	27	Eure	99,8	601843	45	14,12	0,9	-15	-1,2	1
49	Maine-et-Loire	114,1	813493	50	17,58	66	Pyrénées-Orientales	115,2	474452	72	7,59	1,1	22	-10,0	-1
54	Meurthe-et-Moselle	139,9	733481	61	49,90	60	Oise	140,5	824503	44	51,18	0,6	-17	1,3	-1
35	Ille-et-Vilaine	155,3	1060199	74	8,39	84	Vaucluse	156,7	559479	58	7,33	1,4	-16	-1,1	1
84	Vaucluse	156,7	559479	58	7,33	33	Gironde	157,1	1583384	48	10,42	0,4	-10	3,1	-1
57	Moselle	168,2	1043522	51	81,84	38	Isère	168,6	1258722	85	12,16	0,4	34	-69,7	-1
76	Seine-Maritime	200	1254378	45	14,75	44	Loire-Atlantique	200,9	1394909	65	12,19	0,9	20	-2,6	-1
67	Bas-Rhin	235,8	1125559	38	61,04	77	Seine-et-Marne	236,3	1403997	52	51,00	0,5	14	-10,0	-1
77	Seine-et-Marne	236,3	1403997	52	51,00	90	Territoire de Belfort	236,4	142622	137	57,14	0,1	85	6,1	1
90	Territoire de Belfort	236,4	142622	137	57,14	67	Bas-Rhin	235,8	1125559	38	61,04	0,6	-99	3,9	-1

****

Matching criteria : abs(de1-de2)<Δ*max(de1,de2)   Δ=0.01

**Tot sign negatives**	**21**
**Tot sign positives**	**17**

Dep Code: Department Code | **Dep Name**: Name of the French Department | **Dens (km^2)**: Population Density per square kilometer | **Pop**: Total Population | **Radon Avg (Bq/m^3)**: Average Radon Concentration in Becquerels per cubic meter | **Hosp/10^5**: Hospitalization Rate per 100,000 population | **Δ Dens (km^2)**: Difference in Population Density between paired departments (<1%) | **Δ Radon (Bq/m^3)**: Difference in Radon Concentration between paired departments | **Δ Hosp (/10^5)**: Difference in Hospitalization Rates between paired departments | **Sign Consistency**: Indicates if changes in Radon and Density align for matched pairs (positive or negative alignment), Sign[Δrai ×Δdci]
