# Supplementary material for: Radon exposure and COVID-19 mortality in pre-vaccination period: What links might exist?
Source: PLoS One. 2025 Dec 5;20(12):e0337320. doi: 10.1371/journal.pone.0337320 (PMC12680143; doi:10.1371/journal.pone.0337320)
Supplement: S2 Table — This table provides a detailed breakdown of the statistical analysis, exploring the relationship between population density, radon exposure, and the total hospitalization number across various regions in France using matching pair differential. (PDF) [file pone.0337320.s002.pdf]

Dep Code	Dep Name	Dens (km²)	pop	Radon Avg (Bq/m³)	Hosp/10⁵	Dep Code	Dep Name	Dens (km²)	pop	Radon Avg (Bq/m³)	Hosp/10⁵	Δ Dens (km²)	Δ Radon (Bq/m³)	Δ Hosp (/10⁵)	sign cons.
5	Hautes-Alpes	25,4	141284	144	114,0	15	Cantal	25,5	145143	161	42,7	0,1	17	-71,2	-1
55	Meuse	30,4	187187	62	324,3	32	Gers	30,5	191091	66	59,1	0,1	4	-265,1	-1
32	Gers	30,5	191091	66	59,1	58	Nièvre	30,7	207182	115	61,3	0,2	49	2,2	1
58	Nièvre	30,7	207182	115	61,3	55	Meuse	30,4	187187	62	324,3	0,3	-53	263,0	-1
36	Indre	32,9	222232	102	125,5	46	Lot	33,2	173828	88	52,9	0,3	-14	-72,6	1
43	Haute-Loire	45,7	227283	157	55,4	24	Dordogne	45,8	413606	79	27,1	0,1	-78	-28,3	1
24	Dordogne	45,8	413606	79	27,1	89	Yonne	45,9	338291	68	151,1	0,1	-11	124,0	-1
89	Yonne	45,9	338291	68	151,1	3	Allier	46,2	337988	145	82,0	0,3	77	-69,1	-1
3	Allier	46,2	337988	145	74,0	24	Dordogne	45,8	413606	79	27,1	0,4	-66	-46,9	1
43	Haute-Loire	45,7	227283	157	55,4	89	Yonne	45,9	338291	68	151,1	0,2	-89	95,7	-1
65	Hautes-Pyrénées	51	228530	108	83,1	10	Aube	51,4	310020	35	255,1	0,4	-73	172,0	-1
39	Jura	52,1	260188	92	149,1	41	Loir-et-Cher	52,5	331915	70	120,2	0,4	-22	-28,9	1
41	Loir-et-Cher	52,5	331915	70	120,2	8	Ardennes	52,7	273579	95	102,7	0,2	25	-17,5	-1
7	Ardèche	58,8	325712	134	173,5	16	Charente	59,3	352335	90	21,0	0,5	-44	-152,5	1
16	Charente	59,3	352335	90	21,0	53	Mayenne	59,5	307445	96	95,0	0,2	6	74,0	1
53	Mayenne	59,5	307445	96	95,0	11	Aude	59,9	370260	86	80,8	0,4	-10	-14,2	1
11	Aude	59,9	370260	86	80,8	7	Ardèche	58,8	325712	134	173,5	0,5	44	92,7	1
47	Lot-et-Garonne	62,1	332842	69	30,3	86	Vienne	62,4	436876	91	44,4	0,3	22	14,1	1
86	Vienne	62,4	436876	91	44,4	79	Deux-Sèvres	62,5	374351	103	25,1	0,1	12	-19,3	-1
79	Deux-Sèvres	62,5	374351	103	25,1	88	Vosges	62,9	367673	135	44,4	0,4	32	19,3	1
88	Vosges	62,9	367673	135	276,3	86	Vienne	62,4	436876	91	44,4	0,5	-44	-231,9	1
47	Lot-et-Garonne	62,1	332842	69	30,3	79	Deux-Sèvres	62,5	374351	103	25,1	0,4	34	-5,2	-1
80	Somme	92,8	572443	42	174,2	17	Charente-Maritime	93,6	644303	45	31,7	0,8	3	-142,5	-1
37	Indre-et-Loire	98,9	606511	60	78,2	45	Loiret	99,5	678105	55	114,9	0,6	-5	36,7	-1
45	Loiret	99,5	678105	55	114,9	27	Eure	99,8	601843	45	59,5	0,3	-10	-55,4	1
27	Eure	99,8	601843	45	59,5	85	Vendée	99,8	675247	83	48,4	0	38	-11,1	-1
85	Vendée	99,8	675247	83	48,4	45	Loiret	99,5	678105	55	114,9	0,3	-28	66,5	-1
85	Vendée	99,8	675247	83	48,4	37	Indre-et-Loire	98,9	606511	60	78,2	0,9	-23	29,7	-1
37	Indre-et-Loire	98,9	606511	60	78,2	27	Eure	99,8	601843	45	59,5	0,9	-15	-18,7	1
49	Maine-et-Loire	114,1	813493	50	101,8	66	Pyrénées-Orientales	115,2	474452	72	68,5	1,1	22	-33,3	-1
54	Meurthe-et-Moselle	139,9	733481	61	224,0	60	Oise	140,5	824503	44	200,1	0,6	-17	-23,9	1
35	Ille-et-Vilaine	155,3	1060199	74	56,6	84	Vaucluse	156,7	559479	58	56,8	1,4	-16	0,2	-1
84	Vaucluse	156,7	559479	58	56,8	33	Gironde	157,1	1583384	48	76,0	0,4	-10	19,2	-1
57	Moselle	168,2	1043522	51	329,6	38	Isère	168,6	1258722	85	71,2	0,4	34	-258,4	-1
76	Seine-Maritime	200	1254378	45	82,3	44	Loire-Atlantique	200,9	1394909	65	68,0	0,9	20	-14,3	-1
67	Bas-Rhin	235,8	1125559	38	307,0	77	Seine-et-Marne	236,3	1403997	52	254,6	0,5	14	-52,3	-1
77	Seine-et-Marne	236,3	1403997	52	254,6	90	Territoire de Belfort	236,4	142622	137	61,3	0,1	85	-193,3	-1
90	Territoire de Belfort	236,4	142622	137	61,3	67	Bas-Rhin	235,8	1125559	38	307,0	0,6	-99	245,6	-1

****

**Matching criteria : abs(de1-de2)<Δ*max(de1,de2) Δ=0.01**	
**(*) SG = Sign (Δra*Δho)**	

**Tot sign negatives**	**23**
**Tot sign positives**	**15**

Dep Code: Department Code | **Dep Name**: Name of the French Department | **Dens (km²)**: Population Density per square kilometer | **Pop**: Total Population | **Radon Avg (Bq/m³)**: Average Radon Concentration in Becquerels per cubic meter | **Hosp/10⁵**: Hospitalization Rate per 100,000 population | **Δ Dens (km²)**: Difference in Population Density between paired departments (<1%) | **Δ Radon (Bq/m³)**: Difference in Radon Concentration between paired departments | **Δ Hosp (/10⁵)**: Difference in Hospitalization Rates between paired departments | **Sign Consistency:** Indicates if changes in Radon and Density align for matched pairs (positive or negative alignment), Sign[Δrai ×Δdci]
