# Supplementary material for: Radon exposure and COVID-19 mortality in pre-vaccination period: What links might exist?
Source: PLoS One. 2025 Dec 5;20(12):e0337320. doi: 10.1371/journal.pone.0337320 (PMC12680143; doi:10.1371/journal.pone.0337320)
Supplement: Attachment — Submitted filename: Radon and COVID-19 Plosone.pdf [file pone.0337320.s003.pdf]

PLOS ONE

Radon exposure and COVID-19 mortality in pre-vaccination period: what links might exist?

--Manuscript Draft--

**Manuscript Number:**	PONE-D-24-57007
**Article Type:**	Research Article
**Full Title:**	Radon exposure and COVID-19 mortality in pre-vaccination period: what links might exist?
**Short Title:**	Radon exposure and COVID-19 mortality in pre-vaccination period.
**Corresponding Author:**	Stephanie Jonathan, M.D<br>Université Côte d'Azur Faculté de Médecine: Universite Cote d'Azur Faculte de Medecine<br>NICE, Cedex 2 FRANCE
**Keywords:**	Radon exposure; COVID-19 mortality; Indoor radon concentration; Statistical analysis; Pre-vaccination period
**Abstract:**	Radon, a naturally occurring radioactive gas known for its health risks, has recently gained attention for its potential protective role against COVID-19 mortality. This cross-sectional ecological study examined the relationship between indoor radon exposure and COVID-19 mortality rates across eight countries, including several European nations, the United States, and the State of Kerala, India, during the pre-vaccination period. The analyzed data on the subject were derived from recent scientific publications. The environmental aspect was represented by the variable "indoor radon concentration or probability of exceeding a radon concentration in indoor air," depending on data availability. Using national radon surveys and COVID-19 mortality statistics, statistical analyses, including Spearman's correlation and Kendall Tau, were conducted between March and December 2020. The findings revealed a consistent negative correlation between higher radon concentrations and lower COVID-19 mortality rates. Regions such as Finland and Sweden, where radon exposure was relatively high, experienced significantly lower mortality. With Sweden and Finland showing a mortality risk reduction factor of respectively 1,42 and 5,47 during the first wave compared to the UK where Radon levels are very low. Although the findings are not overwhelmingly strong, the data suggest that radon exposure may have a mitigating effect on COVID-19 mortality.
**Order of Authors:**	Jean-François Coudert, PhD
	Ekaterina Dadachova, PhD
	Gilles Maignant, PhD
	Stephanie Jonathan, M.D
**Additional Information:**	
**Question**	**Response**
**Financial Disclosure**<br><br>Enter a financial disclosure statement that describes the sources of funding for the work included in this submission. Review the submission guidelines for detailed requirements. View published research articles from *PLOS ONE* for specific examples.<br><br>This statement is required for submission	The author(s) received no specific funding for this work.

Powered by Editorial Manager® and ProduXion Manager® from Aries Systems Corporation

and **will appear in the published article** if the submission is accepted. Please make sure it is accurate.<br><br>**Funded studies**<br>Enter a statement with the following details:<br>• Initials of the authors who received each award<br>• Grant numbers awarded to each author<br>• The full name of each funder<br>• URL of each funder website<br>• Did the sponsors or funders play any role in the study design, data collection and analysis, decision to publish, or preparation of the manuscript?<br><br>Did you receive funding for this work?	
**Competing Interests**<br><br>Use the instructions below to enter a competing interest statement for this submission. On behalf of all authors, disclose any competing interests that could be perceived to bias this work—acknowledging all financial support and any other relevant financial or non-financial competing interests.<br><br>This statement is required for submission and **will appear in the published article** if the submission is accepted. Please make sure it is accurate and that any funding sources listed in your Funding Information later in the submission form are also declared in your Financial Disclosure statement.<br><br>View published research articles from *PLOS ONE* for specific examples.	The authors have declared that no competing interests exist.

**NO authors have competing interests**<br><br>Enter: *The authors have declared that no competing interests exist.*<br><br>**Authors with competing interests**<br><br>Enter competing interest details beginning with this statement:<br><br>*I have read the journal's policy and the authors of this manuscript have the following competing interests: [insert competing interests here]*<br><br>* typeset	
**Ethics Statement**<br><br>Enter an ethics statement for this submission. This statement is required if the study involved:<br><br>• Human participants<br>• Human specimens or tissue<br>• Vertebrate animals or cephalopods<br>• Vertebrate embryos or tissues<br>• Field research<br><br>Write "N/A" if the submission does not require an ethics statement.<br><br>General guidance is provided below. Consult the submission guidelines for detailed instructions. **Make sure that all information entered here is included in the Methods section of the manuscript.**	N/A<br>This study did not involve the direct recruitment of human participants, such as conducting clinical trials, distributing questionnaires, or collecting tissues, data, or samples for research purposes. The study is an ecological retrospective study based on radon exposure, utilizing existing environmental data rather than data from directly recruited individuals.

Powered by Editorial Manager® and ProduXion Manager® from Aries Systems Corporation

**Format for specific study types**<br><br>**Human Subject Research (involving human participants and/or tissue)**<br>• Give the name of the institutional review board or ethics committee that approved the study<br>• Include the approval number and/or a statement indicating approval of this research<br>• Indicate the form of consent obtained (written/oral) or the reason that consent was not obtained (e.g. the data were analyzed anonymously)<br><br>**Animal Research (involving vertebrate animals, embryos or tissues)**<br>• Provide the name of the Institutional Animal Care and Use Committee (IACUC) or other relevant ethics board that reviewed the study protocol, and indicate whether they approved this research or granted a formal waiver of ethical approval<br>• Include an approval number if one was obtained<br>• If the study involved *non-human primates*, add *additional details* about animal welfare and steps taken to ameliorate suffering<br>• If anesthesia, euthanasia, or any kind of animal sacrifice is part of the study, include briefly which substances and/or methods were applied<br><br>**Field Research**<br><br>Include the following details if this study involves the collection of plant, animal, or other materials from a natural setting:<br>• Field permit number<br>• Name of the institution or relevant body that granted permission	
**Data Availability**<br><br>Authors are required to make all data underlying the findings described fully available, without restriction, and from the time of publication. PLOS allows rare exceptions to address legal and ethical concerns. See the PLOS Data Policy and FAQ for detailed information.	Yes - all data are fully available without restriction

A Data Availability Statement describing where the data can be found is required at submission. Your answers to this question constitute the Data Availability Statement and **will be published in the article**, if accepted.<br><br>**Important:** Stating 'data available on request from the author' is not sufficient. If your data are only available upon request, select 'No' for the first question and explain your exceptional situation in the text box.<br><br>Do the authors confirm that all data underlying the findings described in their manuscript are fully available without restriction?	
**Describe where the data may be found in full sentences. If you are copying our sample text, replace any instances of XXX with the appropriate details.**<br><br>• If the data are **held or will be held in a public repository**, include URLs, accession numbers or DOIs. If this information will only be available after acceptance, indicate this by ticking the box below. For example: *All XXX files are available from the XXX database (accession number(s) XXX, XXX.).*<br>• If the data are all contained **within the manuscript and/or Supporting Information files**, enter the following: *All relevant data are within the manuscript and its Supporting Information files.*<br>• If neither of these applies but you are able to provide **details of access elsewhere**, with or without limitations, please do so. For example:<br><br>*Data cannot be shared publicly because of [XXX]. Data are available from the XXX Institutional Data Access / Ethics Committee (contact via XXX) for researchers who meet the criteria for access to confidential data.*<br><br>*The data underlying the results presented in the study are available from (include the name of the third party*	All relevant data are within the manuscript and its Supporting Information files.

and contact information or URL).<br>• This text is appropriate if the data are owned by a third party and authors do not have permission to share the data.<br><br>* typeset	
Additional data availability information:	Tick here if the URLs/accession numbers/DOIs will be available only after acceptance of the manuscript for publication so that we can ensure their inclusion before publication.

Powered by Editorial Manager® and ProduXion Manager® from Aries Systems Corporation

Manuscript

Click here to access/download;Manuscript;Radon_PLOS.pdf

Radon exposure and COVID-19 mortality in pre-vaccination period: what links might exist?

Jean François Coudert[1], Ekaterina Dadachova[2], Gilles Maignant[3], Stephanie Jonathan[3].

1 C&C IngeScience, Mouans-Sartoux, France
2 College of Pharmacy and Nutrition, University of Saskatchewan, Saskatoon, Canada
3 Laboratoire RETINES(Risques, Epidémiologie, Territoires, Informations, Education et Santé)/Département IRIS/Faculté de Médecine, Université Côte d'Azur, Nice, France

* jfcoudert@ingescience.fr (JFC); ekaterina.dadachova@usask.ca (ED); gilles.maignant@univ-cotedazur.fr (GM); stephanie.jonathan@univ-cotedazur.fr (SJ)

Abstract

Radon, a naturally occurring radioactive gas known for its health risks, has recently gained attention for its potential protective role against COVID-19 mortality. This cross-sectional ecological study examined the relationship between indoor radon exposure and COVID-19 mortality rates across eight countries, including several European nations, the United States, and the State of Kerala, India, during the pre-vaccination period. The analyzed data on the subject were derived from recent scientific publications. The environmental aspect was represented by the variable "indoor radon concentration or probability of exceeding a radon concentration in indoor air," depending on data availability. Using national radon surveys and COVID-19 mortality statistics, statistical analyses, including Spearman's correlation and Kendall Tau, were conducted between March and December 2020. T[illegible] findings revealed a consistent negative correlation between higher radon concentrations and lower COVID-19 mortality rates. Regions such as Finland and Sweden, where radon exposure was relatively high, experienced significantly lower mortality. With Sweden and Finland showing a mortality risk reduction factor of respectively 1,42 and 5,47 during the first wave compared to the UK where Radon levels are very low. Although the findings are not overwhelmingly strong, the data suggest that radon exposure may have a mitigating effect on COVID-19 mortality.

Introduction

One of the first outbreaks of the COVID-19 pandemic that appeared on March 1, 2020, in the department of Morbihan in western France [1] did not result in high mortality numbers, in contrast to the outbreaks which happened simultaneously in eastern France. It might be possible that an environmental factor could help to explain these observations. ^{222}Radon (^{222}Rn), referred to in this paper as radon, is a radioactive noble gas with a physical half-life of 3.8 days. Radon, emitted naturally by the descendants of the Uranium natural, is naturally present in the soil of many territories of the world, including Morbihan in France. It concentrates in basements and floors of homes and buildings and spreads through the various ventilation systems. It can also be emitted directly by the construction materials used or by the beach sands used in the cements [2, 3]. The significant body of published data related to thermal spas shows the

therapeutic benefits of controlled exposure to radon for treating inflammatory diseases such as rheumatoid arthritis [4, 5]. Recent publications on COVID-19 [6, 7] show that exposure to another type of ionizing radiation, i.e., X-rays, resulted in impressive curative effects in COVID-19 patients.

Based on these data, we hypothesized that exposure to radon through inhalation at home (indoor) could constitute an environmental factor contributing to resistance against COVID-19. To evaluate the hypothesis that indoor radon exposure may result in reduced mortality of COVID-19, we performed an exploratory ecological study, where we analyzed aggregated data at the population level and identified statistically significant correlations (Spearman, Kendall Tau) between two parameters: ^{222}Radon (^{222}Rn) concentration per m^3 in the surroundings of residential buildings and the mortality rate of the COVID-19-infected population during the pandemic's pre-vaccination period. It is important to note that our observations relate only to COVID-19, which is an acute medical condition, and are in no way contradicting the correlations between radon exposure and lung cancer, which evolves decades after exposure.

This study is divided into four parts to explore the relationship between indoor radon exposure and COVID-19 mortality rates comprehensively. The first part qualitatively compares mortality rates and their trends in different European countries where indoor radon exposure is prominent. The second part explores the epidemiological data from Kerala, India, known for having some of the highest levels of radon exposure worldwide (High Background Natural Radioactive Areas-HBNRAs) [8]. The third part compares the average radon exposure limits previously accepted by health authorities with short-term radon exposure levels, whether or not they appear for therapeutic purposes in the world's numerous thermal baths, some of which have existed since ancient times. The final part is devoted to a statistical correlational study of COVID-19 mortality data from French departments and continental states in the USA. In conclusion, indoor radon exposure affects COVID-19 mortality and appears to be statistically valid based on the analysis conducted.

Current definition of radon exposure

Exposure is defined as the concentration of radioactivity in the air we breathe. It is measured in Bequerels per cubic meter (Bq/m^3 in the SI system) or in PicoCurie per liter (pCi/L) in the traditional system (1 PicoCurie/L = 37 Bq/m^3). One Bq corresponds to one disintegration (radiation emission) of the radio-element per second. Radon radiation consists of alpha particles, which are He nuclei with 2 protons and 2 neutrons. Throughout this article, we will refer to indoor radon and its progeny, present in homes, as radon.

Radon, which is recognized as a carcinogen by the United Nations Scientific Committee on the Effects of Atomic Radiation (UNSCEAR) [9], is widely measured in indoor air, and most countries have defined exposure limit values above which remedial measures should be taken. The World Health Organization (WHO), the European Commission, and the Institut de Radioprotection et de Sûreté Nucléaire (IRSN) in France recommend a limit value of 300 Bq/m^3 averaged over one year of exposure [10]. In the USA, it is considered that residential buildings require remediation if values exceed 4 pCi/L (148 Bq/m^3) [11].

Indoor radon measurements

The natural disintegration of uranium often leads to the accumulation of radon on the ground floors of dwellings through natural or mechanical ventilation systems (MVS). Geological, geophysical, and climatological variables interact in complex ways to

influence this process. This complexity is described in the USGS/EPA Radon Potential Assessment Open File Report 93-292 [12], the European Radon Mapping Project [13], which compiles data from various European countries, and the French Atlas Radon [14]. Fig 1 [13] illustrates the complexity of these phenomena.

Fig 1. Description of the phenomenon involved in the risk evaluation of the indoor radon. Adapted from Bossew P. The European radon mapping project. Radiat Prot Dosimetry. 2013;157(3):522–530.

There are more than 100,000 home measurements available in the USA, 818,704 in Europe, and 12,641 in France. The indoor radon concentrations from these sources result from various processes, including measurement density per geographical entity, measurement methods, data compilation, and processing. This issue is discussed in detail in the European Project for the different countries [15]. For example, measurements in the USA use charcoal canisters in the lowest living area for two to seven days, while in France, measurements are taken over two months, mostly during the cold season, to account for climatological factors. The detailed procedures, data, and sources for each country's radon map (Fig 2) are provided in a European Commission-JRC publication [16]. The number of dwellings monitored up to 2005 is listed in Table 1.

Fig 2. Map of indoor radon in Europe, September 2018. Adapted from European Commision, Joint Research Centre (JRC), Directorate G -Nuclear Safety Security, REM project. Arithmetic means over 10 x 10 km cells of long-term radon concentration in ground-floor rooms.

Table 1. Number of dwellings monitored up to 2005 for each countries of the radon map.

Country	Number of dwellings monitored	Country	Number of dwellings monitored
Albania	110	Latvia	300
Austria	16000	Lithuania	400
Belgium	9000	Luxembourg	2619
Croatia	782	Malta	90
Cyprus	84	Netherlands	1846
Czech Republic	150000	Norway	51925
Denmark	3120	Poland	4098
Estonia	515	Portugal	3317
Finlande	73074	Romania	567
France	12261	Serbia-Montenegro*	968
FYROM	NA	Slovakia	4019
Germany	>50000	Slovenia	2512
Greece	1277	Spain	5600
Hungary	15602	Sweden	500000
Ireland	11319	Switzerland	55000
Italy	5361		

* Province of Vojvodina only.

All of these measurement campaigns have mobilized many experts in different countries over several years. They have been the subject of numerous international scientific exchanges and debates. These measurements are used as criteria for enforcing regulations and laws in various state governments. To our knowledge, they represent the best available data for this research. An individual's radon exposure also depends on

sources beyond the local geology of their dwelling. These sources include exhalation from building materials that originate from other regions or countries [17].

Comparison of mortality rates and exposure to indoor radon

To establish this comparison, we analyzed the evolution of the average deaths per 100,000 inhabitants—referred to as the "mortality rate"—from the beginning of the pandemic (mid-January 2020 to mid-December 2020) in countries with particularly high radon concentrations to derive a relevant indicator. The number of infections and recoveries cannot be used due to their dependence on the number of tests conducted. The study period concludes with the start of the vaccination campaigns.

The European Commission's Joint Research Centre (JRC) publishes the European Atlas of Natural Radiation, which maps indoor radon measurements in all European Union states [18]. This map is continuously updated and is created using a 10 km x 10 km grid (Fig 2), allowing for the identification of territories with high radon concentrations. To utilize this information, we focus on territories where mortality data related to the COVID-19 pandemic is available. This excludes areas at the intersections of several countries or political regions, such as the eastern part of France-Switzerland-Italy-Northern Austria, or areas that are too small, where COVID-19 data is not specifically geolocated or published. The United Kingdom, Sweden, and Finland were selected for this analysis.

The mortality curves for these three countries, calculated using data from the European Center for Disease Prevention and Control (ECDC), show significantly different mortality rates for the period from January 1, 2020 (week 1) to February 22, 2020 (week 59) (Fig 3).

Fig 3. Comparison of mortality rates for the United Kingdom, Sweden and Finland.

This figure shows that throughout this period, Finland and Sweden exhibit higher radon exposure and lower mortality rates compared to the United Kingdom, which has minimal radon exposure (except in Scotland, Wales, and Cornwall).

To complete the comparison between these three countries, we collected the maximum mortality rates during the first and second waves of the pandemic, as shown in Table 2. Radon levels, measured as the Activity Median concentration in the air and expressed in becquerels per cubic meter (AM_Bq/m^3), are also reported for each country to provide context for environmental differences. We calculated the mortality rate ratio using the United Kingdom (0-50 AM_Bq/m^3) as the reference country. We compared this data with Sweden (50-200 AM_Bq/m^3) and Finland (50-1000 AM_Bq/m^3), both of which have significantly higher radon levels.

Table 2. Comparison of the Peak Values (PV ie maximum) of the weekly deaths per 100,000 inhabitants between United Kingdom, Sweden and Finland during the 1st and the 2nd wave of the pandemics.

Country	Peak Value	1st Wave	*MR	2nd Wave	MR
**United Kingdom**	Year-week n°	2020-15		2021-03	
	Value	9.46	-	12.75	-
**Sweden**	Year-week n°	2020-15		2022-53	
	Value	6.64	0.70	6.43	0.50
**Finland**	Year-week n°	2020-17		2021-01	
	Value	1.73	0.18	0.58	0.05

*Mortality Pick-value Ratio
Source: ECDC

The analysis reveals significant differences in mortality rates correlated with varying levels of radon exposure. Specifically, in Sweden, the presence of radon without barrier or containment measures leads to a notable decreased in mortality risk by 30%, with a mortality pick value ratio of 0.70 (6.64/9.46) observed during the first wave of the pandemic. This impact becomes even more pronounced when barrier and containment measures are implemented, as seen in Finland, where the mortality pick value ratio reduced substantially to 82%, with a mortality pick value ratio 0.18 (1.73/9.46).

Overall, the analysis of mortality rates in other countries with elevated radon exposure demonstrates consistent findings, indicating a clear suggestion of association between increased radon levels and lower mortality rates during the pandemic. This suggests that addressing radon exposure may be critical in mitigating health risks associated with COVID-19.

Several countries experienced virtually no first wave of mortality. To illustrate this point, we can analyze the case of the Czech Republic (Fig 4). Radon concentration is high and well distributed within its borders, as shown on the JRC map. The first COVID-19 case appeared on March 1, 2020, as in other European countries. However, the Czech Republic was not affected by the first wave, similar to other high radon-exposed countries like Sweden, Finland, and Kerala. Public policy regarding population protection in Czechia has been inconsistent, with a population not motivated by protective measures (e.g., barrier gestures, masks). This resulted in a first wave that occurred later when other European countries were already hit by the second wave.

The magnitude of the waves in Czechia was likely driven by spikes in infections due to this context. The result is striking, as shown in Fig 4. There was nearly no mortality during the first wave, with a rate of 0.66 for week 15, comparable to Finland's rate of 1.73. Other countries with high radon exposure, as shown on the Joint Research Center (JRC) map, also did not experience a significant first wave. These include Slovakia (Fig 4), with a mortality rate of 0.18 for week 16, and Estonia (Fig 4), where radon is prevalent on the most populated coast (Tallinn region), with a mortality rate of 1.3 in week 16. These countries exhibit similar mortality curves.

Outside Europe, data are available for Georgia (Fig 4), although the radon census there is incomplete, and it does not appear on the JRC map. Local studies [19] show areas of high radon concentration, particularly in the capital, Tbilisi, where the mortality rate remained very low.

Fig 4. Mortality rate for the UK, Czech Republic, Slovakia, Estonia, and Georgia.

Study of High Background Natural Radioactive Areas (HBNRAs)

There are areas in the world where radon emissions are extremely high. These areas are identified under the name of "High Background Natural Radioactive Areas (HBNRAs)." They are regularly the subject of epidemiological studies concerning the frequency and nature of cancers in exposed populations, without any definitive conclusions being drawn as to the impact on the number of induced cancers [20]. It is interesting to study these areas in relation to COVID-19. Four well-identified areas include the Ramsar region in Iran on the Caspian Sea, the city of Guarapari in Brazil on the Atlantic coast, the city of Yangjiang in China on the South China Sea (southwest of Hong Kong), and the state of Kerala in India on the southwest coast of the Arabian Sea.

The natural radioactivity of the first three sites is rather localized (hot springs, beaches), making it difficult to draw conclusions regarding the pandemic. In particular,

the extent of the use of radioactive beach sand for construction is not well known. However, the state of Kerala, which has a surface area of 38,852 km^2, a population of 35 million, and a density of more than 860 inhabitants per km^2 (compared to 324 in India and 413 in the Netherlands), shows interesting results. The first three COVID-19 cases in India appeared in Kerala in early February 2020, in three different districts, among students returning from Wuhan, China. The state government quickly implemented containment measures, but this did not prevent the disease from spreading rapidly throughout India.

The Health and Family Welfare Department of Kerala set up a daily bulletin with exemplary monitoring of the disease's spread (identification of deaths on a case-by-case basis, age, sex, date, origin of infection, and presence of comorbidities). As shown in Fig 5, the evolution of the number of deaths per week is very low and comparable to that of Finland (Figs 3 and 4).

Fig 5. Mortality rate for the State of Kerala in India. Source: https://dashboard.kerala.gov.in/

Fig 6 shows the impressive results of the pandemic in Kerala, including confirmed cases, recoveries, and the low number of deaths. This suggests the potentially curative effect of radon by reducing deaths in severe cases, rather than preventing the number of severely ill patients.

Fig 6. Evolution of the pandemic in the state of Kerala (what period?), India. Source: https://dashboard.kerala.gov.in/

Materials and methods

Statistical study using France data

To quantify the relationship between radon exposure (Fig 7) and the mortality rate, a statistical study was conducted in France (Table 3) on the correlation between this rate in each metropolitan department (90 departments) (source: Institut National de la Statistique et des Études Économiques - IN[illegible]) and the average radon concentrations in these departments [14].

Table 3. Mortality Rates and Radon Concentrations by France Department for the period of March to Decembre, 2020.

Departments	Mortality rate	Radon conc. (Bq/m^3)	Departments	Mortality rate	Radon conc. (Bq/m^3)
Ain	16.5	55	Lozère	1.3	264
Aisne	53.9	62	Maine-et-Loire	17.6	50
Allier	12.1	145	Manche	10.1	74
Alpes-de-Haute-Provence	7.9	52	Marne	48.2	41
Hautes-Alpes	14.2	144	Haute-Marne	51.2	136
Alpes-Maritimes	17.8	37	Mayenne	15.6	96
Ardèche	35.6	134	Meurthe-et-Moselle	49.9	61
Ardennes	21.9	95	Meuse	55.0	62
Ariège	1.3	129	Morbihan	12.8	142
Aube	44.2	35	Moselle	81.8	51

Mortality Rates and Radon Concentrations by France Department for the period of March to Decembre, 2020 (Continue).

Departments	Mortality rate	Radon conc. (Bq/m^3)	Departments	Mortality rate	Radon conc. (Bq/m^3)
Aude	15.9	86	Nièvre	13.5	115
Aveyron	8.6	101	Nord	26.1	36
Bouches-du-Rhône	28.9	32	Oise	51.2	44
Calvados	12.1	118	Orne	15.5	94
Cantal	6.2	161	Pas-de-Calais	22.0	40
Charente	3.7	90	Puy-de-Dôme	6.9	146
Charente-Maritime	7.9	45	Pyrénées-Atlantiques	4.0	57
Cher	27.9	79	Hautes-Pyrénées	11.8	108
Corrèze	15.3	217	Pyrénées-Orientales	7.6	72
Côte-d'or	47.0	76	Bas-Rhin	61.0	38
Côtes d'Armor	6.7	110	Haut-Rhin	108.8	58
Creuse	12.6	262	Rhône	36.3	99
Dordogne	3.4	79	Haute-Saône	32.5	101
Doubs	28.2	178	Saône-et-Loire	36.5	116
Drôme	25.0	36	Sarthe	15.5	44
Eure	14.1	45	Savoie	17.4	114
Eure-et-Loir	33.5	57	Haute-Savoie	21.2	56
Finistère	4.8	144	Paris	82.7	22
Corse-du-Sud	30.5	263	Seine-Maritime	14.7	45
Haute-Corse	6.2	133	Seine-et-Marne	51.0	52
Gard	13.3	84	Yvelines	37.9	30
Haute-Garonne	6.1	32	Deux-Sèvres	5.9	103
Gers	12.0	66	Somme	40.7	42
Gironde	10.4	48	Tarn	5.9	50
Hérault	10.8	32	Tarn-et-Garonne	2.7	67
Ille-et-Vilaine	8.4	74	Var	13.6	54
Indre	36.9	102	Vaucluse	7.3	58
Indre-et-Loire	15.3	60	Vendée	6.4	83
Isère	12.2	85	Vienne	9.2	91
Jura	23.1	92	Haute-Vienne	6.7	204
Landes	3.2	26	Vosges	72.6	135
Loir-et-Cher	19.6	70	Yonne	28.1	68
Loire	32.5	232	Territoire de Belfort	51.1	137
Haute-Loire	7.9	157	Essonne	42.8	45
Loire-Atlantique	12.2	65	Hauts-de-Seine	69.3	34
Loiret	15.3	55	Seine-St-Denis	63.0	34
Lot	12.7	88	Val-de-Marne	88.7	46
Lot-et-Garonne	3.6	69	Val-D'Oise	59.5	41
Date	01/09/2020	P-value	0.0054	SPEARMAN CORREL.	-0.281

Source: data.gouv.fr

We find in Table 4 a quasi-constant correlation coefficient (-0.27) for random dates between 11/06/20 and 30/10/20, indicating that there are fewer deaths in areas with higher radon exposure (negative correlation coefficients). This correlation progressively

Fig 7. Map of the average concentration of indoor radon measured by department. Source: Institut de Protection et de Sûreté Nucléaire (IPSN) 2000, edited by the Commission de Recherche et Information Indépendantes sur la Radioactivité (CRIIRAD), 2016.

disappears by 12/12/20 in France (-0.137) as the pandemic grows. This could be explained by the fact that the population exposed to indoor radon in each territory remains relatively stable over these periods (i.e., the number of people living in homes exposed to radon) while the number of contamination cases—and conseq[illegible]ly deaths—continues to increase. This observation applies to other countries as well.

Table 4. Spearman correlation coefficients between average radon concentrations and mortality rates in France for 4 different dates in 2020.

France					
**Date (dd/mm/yyyy)**	11/06/2020	17/08/2020	01/09/2020	30/10/2020	12/12/2020
**Spearman Correlation Coef.**	-0.278	-0.280	-0.281	-0.263	-0.137
**P-value**	0.006	0.006	0.006	0.010	0.184

Statistical study from the United States of America

The mortality rates were obtained from data available on the Johns Hopkins University website [21]. In the early 1990s, the U.S. Geological Survey conducted a comprehensive radon survey for all states [12]. This study was based on detailed geological mapping, campaigns measuring uranium in the soil, and thousands of measurements taken from homes selected according to specific criteria, such as the type of dwellings, occupation types, statistical analysis, and the quality and precision of the measurements [22]. We, therefore, used the results of this study to perform correlation calculations. An example of the measurements conducted in the state of New Mexico based on each county is shown in Table 5.

However, the methodology for compiling data at the state level is different from that used for French departments. We have a zoning map at this level (State/EPA Residential Radon Survey Screening Measurements), which shows the estimated percentage of houses with measured radon levels above 4 pCi/L (Figure 8). The results are divided into five classes, numbered from 1 to 5, representing percentages between 0-5%, 5-10%, 10-15%, 15-20%, and more than 20%. This is depicted on a map of the continental US plus Alaska, showing the percentage of homes with a screening level above 4 pCi/L.States are classified into 5 categories according to the ”Estimated Percentage of Dwellings with Radon Levels Detected Above 4 pCi/L”. States are assigned into 5 percentage classes [0-5], [5-10], [10-15], [15-20], [>20] pCi/L (Fig 8).

Fig 8. USA - Percentage of homes exposed to radon levels above 4 pCi/L. Source: USGS-EPA Open-File Report 93-292

Instead of having a mean value for each state, as for the French departments, we have percentage classes for each state. To compare these results with those obtained for France, we calculated the Spearman correlation for four randomly selected dates (Tables 6 and 7). The states of New York and New Jersey were excluded from this analysis. Both states have the highest mortality rates of all states. New York State has 62 counties, and a detailed analysis shows that New York City has 5 counties and Greater New York has 8 more. Of these 13 counties, the most populous—comprising 12 million of the state's 22 million people— are all in class 1 (the lowest % radon exposure), while

Table 5. Screening indoor radon data from the EPA/State Residential Radon Survey of New Mexico conducted during 1988-89.

County	*Meas. total	MEAN	**GEOM. MEAN	MEDIAN	SD	MAX	%>4 pCi/L	%>20 pCi/L
BERNALILLO	406	3.7	2.7	2.6	3.5	27.0	28	1
CATRON	16	1.4	1.0	1.0	1.2	4.2	6	0
CHAVES	52	2.7	2.2	2.3	1.7	6.6	17	0
CIBOLA	6	2.3	1.8	2.3	1.5	4.7	17	0
COLFAX	91	6.0	3.8	3.9	11.5	105.4	49	3
CURRY	47	2.6	1.9	2.1	2.1	11.3	13	0
DEBACA	12	1.3	1.1	1.0	1.0	4.2	8	0
DONA ANA	86	1.8	1.4	1.3	1.4	9.0	7	0
EDDY	51	2.0	1.2	1.3	1.9	7.5	16	0
GRANT	60	2.1	1.3	1.5	2.1	13.4	10	0
GUADALUPE	8	1.3	1.0	1.1	0.8	2.7	0	0
HARDING	12	1.9	1.2	1.1	1.9	6.9	8	0
HIDALGO	18	3.7	2.8	3.4	2.8	12.5	39	0
LEA	50	1.6	1.1	1.1	1.4	7.6	6	0
LINCOLN	18	2.6	1.9	1.7	2.5	10.1	11	0
LOS ALAMOS	42	3.0	2.4	2.7	2.2	13.0	24	0
LUNA	49	3.8	2.5	2.4	4.6	27.7	22	2
MCKINLEY	53	6.0	2.8	3.2	13.0	87.3	34	6
MORA	17	4.6	3.5	3.9	3.2	11.5	41	0
OTERO	46	2.7	1.6	1.9	3.4	21.6	17	2
QUAY	10	3.2	2.7	2.6	1.8	6.0	30	0
RIO ARRIBA	72	3.4	2.3	2.2	4.0	24.7	21	1
ROOSEVELT	44	2.2	1.7	1.7	1.7	7.4	11	0
SAN JUAN	196	2.4	2.0	1.9	2.2	24.8	11	1
SAN MIGUEL	78	4.9	3.1	3.2	5.9	36.2	45	4
SANDOVAL	76	4.6	2.3	2.0	10.2	76.7	20	3
SANTA FE	73	4.6	3.2	3.5	3.8	21.6	41	1
SIERRA	41	1.3	1.0	1.0	0.9	3.9	0	0
SOCORRO	41	2.5	1.9	2.0	1.7	7.2	17	0
TAOS	47	6.3	3.8	4.7	6.6	31.4	57	4
TORRANCE	10	3.9	2.4	2.8	3.6	9.4	50	0
UNION	32	3.4	2.5	2.1	3.1	15.1	31	0
VALENCIA	25	1.9	1.8	1.7	0.8	3.6	0	0

Data represent 2-7 days of charcoal canister measurements from the lowest level of homes tested.*Total number of measurement.**Geometric means
Source: USGS-EPA Open-File Report 93-292-F-p.104

the entire state is in class 5 (the highest). The situation is similar in New Jersey, particularly in Jersey City, where 13 out of 21 counties are part of Greater New York with similar heterogeneity. A detailed analysis of these two states remains to be done.

The variable "radon" is presented as the percentage of exceedance of a reference value (in this case, 4 pCi/L), it represents a categorical ordinal variable which does not necessarily follow a normal distribution, so we turned to a Kendall rank correlation. The principle is the following: a first series is sorted and the ranks of the values of the second series are put in comparison with the first. For each observation, we note the number of subsequent values (noted B in Table 6) that are higher (+1) and lower (-1) than it (noted C in Table 6); this gives a new series of numbers that are balances between positive and negative numbers (S=C-B). All possible ordering of the

Table 6. Spearman correlation detailed data and calculation for 19/09/20 (excluding New York and New Jersey).

**USA States**	**State Code**	***MR**	****Radon map classes**	**USA States**	**State Code**	***MR**	****Radon map classes**
Alaska	AK	5.74	2	California	CA	38.02	1
Vermont	VT	9.30	4	Arkansas	AR	39.13	2
Maine	ME	10.34	5	Ohio	OH	39.46	5
Wyoming	WY	12.27	5	Iowa	IA	40.09	5
Oregon	OR	12.49	3	New Mexico	NM	40.39	5
Utah	UT	13.72	4	Nevada	NV	49.61	3
Montana	MT	14.60	5	Alabama	AL	49.70	2
West Virginia	WV	17.19	5	Indiana	IN	52.03	5
Kansas	KS	20.46	5	Texas	TX	52.16	1
Wisconsin	WI	21.45	5	Georgia	GA	60.75	2
South Dakota	SD	22.61	5	Florida	FL	61.86	1
Nebraska	NE	23.62	5	South Carolina	SC	61.92	1
Oklahoma	OK	23.83	1	Pennsylvania	PA	62.71	5
Idaho	ID	24.68	5	Delaware	DE	63.77	3
Kentucky	KY	25.45	4	Maryland	MD	64.11	4
North Dakota	ND	25.59	5	Illinois	IL	68.56	4
Washington	WA	27.97	2	Michigan	MI	69.79	2
Missouri	MO	31.04	4	Arizona	AZ	75.16	2
North Carolina	NC	31.08	2	Washington D.C	DC	87.71	2
Tennessee	TN	32.11	4	Mississippi	MS	94.38	1
New Hampshire	NH	32.21	5	Rhode Island	RI	102.70	1
Virginia	VA	35.03	3	Louisiana	LA	114.87	1
Colorado	CO	35.11	5	Connecticut	CT	125.99	1
Minnesota	MN	35.73	5	Massachusetts	MA	134.86	5
**Date**	**19/09/2020**		**SPEARMAN CORRELATION**			**-0.440**	

*Deaths for 100.000 inhabitants; **USGS-EPA indoor Radon map classes (Fig.8). Source: github-nytimes and USGS-EPA Open-File Report 93-292-F

Table 7. Spearman correlations for four successive randomly selected dates (excluding New York and New Jersey).

**USA**				
**Date (dd/mm/yyyy)**	07/01/2020	19/08/2020	19/09/2020	30/11/2020
**Spearman Correlation Coef.**	-0.165	-0.406	-0.440	-0.258
**P-value**	0.261	0.004	0.002	0.076

Source: github-nytimes and USGS-EPA Open-File Report 93-292-F

[radon-class] pairs are considered i.e. binomial coefficient $\binom{n}{2}$. The Kendall correlation coefficient called Tau (τ) is then calculated with the following formula:

$$\tau = \frac{S}{\frac{n(n-1)}{2}} = \frac{2S}{n(n-1)} \qquad (1)$$

τ varies between -1 and 1, it can be interpreted as a Spearman correlation coefficient, i.e. the closer it is to 1 (respectively -1), the more certain we are that there is a positive correlation, i.e. a variation in the same direction between the two variables (respectively negative). If is close to 0, the probability that there is no monotonic relationship between the two variables [mortality rate-indoor radon] is high.

In the case where there are ties, a variant of the Kendall correlation called τ-b (τb)

is used, whose formulations are somewhat more complex [23]. From the map of the percentages of excess of the 4 pCi/L threshold of radon, we created a table allowing to assign to each state of the USA, one of the five predefined classes, and put opposite the mortality rates [21], classified in alphabetical order (from Alabama to Wyoming). A sorting on the Mortality Rate column is performed, the states are then ranked according to an increasing rate. The Kendall correlation coefficient is calculated between the two columns Mortality Rate and Radon Map (Table 6) Classes. This correlation is tested for different arbitrary dates among the available data (01 July 2020, 19 August 2020, 19 September 2020, 30 November 2020). The following data analysis was generated using documentation from "Real Statistics Resources Pack software" Copyright (2013-2021), Zaiontz C [24]. The "Kendall's Correlation Testing with Ties" formulas provided for Excel were implemented and tested by us in Libre Office V7.1.3.2 (x64).

The Kendall correlation calculations were performed (Table 8) for the 4 same dates: 01/07/2020 at the end of the first wave in the USA, 19/08/2020, 19/09/2020 during the plateau that followed, and 30/11/2020 during the rise of the second wave and before the start of vaccinations.

Table 8. Tau-b and p-value calculations for all U.S. states excluding New York and New Jersey for four selected dates.

**Kendall Tau-b USA 48 États Radon Zones from Table 1: %>4pCi/L**				
	**01/07/20**	**19/08/20**	**19/09/20**	**30/11/20**
n	48	48	48	48
C(n,2)	1128	1128	1128	1128
B	486	582	588	526
C	349	267	260	328
Tau-b	-0.141	-0.322	-0.335	-0.202
std error	0.0997	0.0997	0.0997	0.0997
z	-1.4132	-3.22883	-3.36057	-2.02321
Z-crit	1.40507	2.80703	2.74778	1.95996
P-value	0.1576	0.0012	0.0008	0.0431

Source: Zaiontz C.(2020), Real Statistics Using Excel. www.real-statistics.com, implemented in LibreOffice 7.1.3.2 (x64)

For sufficiently large samples (n>10), Tau follows a normal distribution, allowing for statistical evaluation of the results. Examination of the data in Table 8 revealed that the probabilities (p-values) were sufficiently low (<0.05) after 19/08/20 to conclude that the correlations presented for the U.S. are statistically significant. It is also noted that as the epidemic waves increase, the correlation weakens, as explained previously for France.

Thus, there is strong convergence of observations on independent data, suggesting that year-round exposure to indoor radon and its progeny results in lower mortality from COVID-19.

Discussion

In this section, we discuss two aspects. The first relates to the statistical processing of French data to support the previous results, the second to more general considerations on the impact of radon.

Statistical Processing of French Data

In observational epidemiological studies, matching methods are extensively used to estimate causal effects between exposures and outcomes to control for potential confounders and minimize bias [25]. The development of these methods is based on the

causal effect concept, which is mathematically defined as the difference in the outcome under exposure for the same individual or unit (Yi=Yi(1) Yi(0)). These methods highlight areas where the covariate distribution lacks sufficient overlap between treatment and control groups, thus preventing over-reliance on extrapolation for exposure effect estimates [26]. Rubin and Thomas [27] demonstrated that analytic approximations for bias reduction in a linear combination of covariates, including outcomes, remain effective even when the assumption of normal distribution is violated. This formulation applies to aggregate data, such as schools or communities, and in this study, to regions.

We noted that regions in the USA and France with higher indoor radon levels often had lower population densities due to geography. To investigate this further, we carried out an analysis on French data, comparing 2 by 2 departments whose population densities are close to within 1%.

Two departments dpt1, dpt2 with respective densities de1 and de2, form a Ci pair if :

$$\Delta \mathrm{de}_i = |\mathrm{de}_1 - \mathrm{de}_2| < 0.01 \max(\mathrm{de}_1, \mathrm{de}_2) \tag{2}$$

We thus obtained 38 Ci pairs among the 95 French départements. We observe that the members of these pairs are localized far from each others in France so that population moves are unlikely. For each of these pairs, we calculated the difference in indoor radon levels ra1 and ra2, the difference in mortality rates dc1 and dc2, and the difference in hospitalization rates ho1 and ho2. We multiplied these differences to calculate the signs of them ($\mathrm{score}_{\mathrm{dci}}$ and $\mathrm{score}_{\mathrm{hoi}}$):

$$\Delta \mathrm{ra}_i = (\mathrm{ra}_1 - \mathrm{ra}_2)_i, \quad \Delta \mathrm{dc}_i = (\mathrm{dc}_1 - \mathrm{dc}_2)_i, \quad \Delta \mathrm{ho}_i = (\mathrm{ho}_1 - \mathrm{ho}_2)_i \tag{3}$$

$$\mathrm{score}_{\mathrm{dc}i} = \mathrm{Signe}\left[\Delta \mathrm{ra}_i \cdot \Delta \mathrm{dc}_i\right], \quad \mathrm{score}_{\mathrm{ho}i} = \mathrm{Signe}\left[\Delta \mathrm{ra}_i \cdot \Delta \mathrm{ho}_i\right] \tag{4}$$

If the score is negative, this means that, for the pair considered, the higher the indoor radon level, the lower the mortality or hospitalization rates.

The results are provided in Table S1 and S2 in the supporting information section. For mortality rates, there are 21 pairs with negative scores and 17 with positive scores. For hospitalization rates, there are 23 pairs with negative scores and 15 with positive scores, indicating a stronger trend for hospitalization rates compared to mortality rates. These findings suggest a consistent effect of indoor radon exposure on health outcomes (mortality and hospitalization rates) among départements with very similar population densities. This observation more pronounced for hospitalization rates (referring to individuals leaving their homes) than for mortality rates (pertaining to individuals already hospitalized).

Radon Activity

Since ancient times, certain geological sites have been identified for their curative properties for various diseases. Many of these sites are still active today as spas, attracting tens of thousands of people per year worldwide and constituting a significant field for medical and wellness treatments. Many resorts now claim, in brochures explaining their therapies, medically controlled exposure to radon in several forms (inhalation, baths, etc.) as a health benefit for treating inflammatory rheumatoid arthritis.

Despite ongoing discussions among experts, it is recognized through the work of Franke A. [5] that radon exposure-based spa therapy is covered by different health

systems in several European countries (Germany, Austria) as noted by Kabat G. [28]. A study on the concentrations of radon to which patients are exposed—just a few hours per day, over a few weeks, for one or more consecutive years—provides useful insights. One can examine the data available from thermal spas, where radon emanations, whether claimed or not, are almost always present. Améon R. [29] conducted a detailed study on this subject, as shown in Table 9.

Table 9. Radon activity by volume (Av) in the air, measured in various spa rooms.

**Rooms (Locaux)**	**A_v (Bq/m^3)**	**Note**	**Spa town**	**Reference**
Administrative building	<40-410		Spain	Soto et Gomez (1999)
	30-220		Bad Gastein (Austria)	Lettner et al. (1996)
Technical tunnel	>100 000			Schmitz et Frische (1993)
Fountains hall	280	Relaxation room	Bad Münster (Germany)	Sansoni (1998)
	1491		Misasa (Japan)	Morinaga et al. (1984)
Pools hall	4 300-7 000	Day-night	Rudas (Hungary)	Szerbin (1996)
	22000		Bagnères-de-Luchon (France)	Grandpierre et al. (1962)
Aero baths	1300	Without ventilation	Evaux-les-Bains (France)	Améon et al. (2000)
	150	With ventilation		Améon et al. (2001)
	40-5 200	Different locations	Spain	Soto et Gómez (1999)
	40-5 000	Outside-with treatments	Radenci (Yugoslavia)	Kobal et Renier (1987)
Spa tunnels	16 600		Bad Gastein (Austria)	Uzunov et al. (1981)
	2700		Misasa (Japan)	Morinaga et al. (1984)
Spa caves	20000	Summer value	Hospital (Hungary)	Szerbin (1996)
	14000	Summer value	Bagnères-de-Luchon (France)	Grandpierre et al. (1962)
Steam room	7500		Evaux-les-Bains (France)	Améon et al. (2000)
	103000	Inhalation masks (radon therapy)	Las Caldas (Spain)	Soto et al. (1995)
Inhalation room	12 8000		Bad Münster (Germany)	Sansoni (1998)

Source: Ameon R. -2003

It can be seen that in Bad Münster, Germany (a spa covered by the German health system), the concentration in the inhalation room is 128 kBq/m^3. In the thermal galleries of Bad Gastein, Austria (a spa covered by the Austrian health system), the concentration is 166 kBq/m^3. In Bagnères de Luchon, France (where radon exposure is not claimed in the therapy), it is 14 kBq/m^3 in the spa caves.

What are the mechanisms behind radon's effects? The first possible explanation is the anti-inflammatory effect of low-dose ionizing radiation. In this regard, several recent studies have followed up on research conducted as far back as the 1940s, which explored the use of low-dose X-ray irradiation of the lungs to treat pneumonia [30–34]. Recent publications have reported success rates ranging from 10% to 30%. However, with the advent of antibiotics, which are much more effective and easier to use, this line of research was abandoned. Despite ongoing debate, these studies highlight the potential of nuclear radiation to mitigate the effects of COVID-19. One study by Clayton B. Hess [35] from Emory University Hospital, Atlanta, reported that in a cohort of 10 elderly hospitalized COVID-19 patients who were oxygen-dependent, seven patients recovered to room air in a median of 3 days, and were discharged in a median of 12 days. An article by Mortazavi [7] provides a comprehensive review of the results of

low-dose radiation therapy (LD-RT) on COVID-19 patients. Taken together, the anti-inflammatory effects of low-dose radiation are comparable to those of steroid hormones given to many COVID-19 patients, but without the severe immunosuppressive effects seen with the latter [36].

Another possible mechanism for radon's effect could be the destruction of virions by alpha particles emitted during the decay of ^{222}Rn. To understand this, it's essential to compare the number of SARS-CoV-2 virions present in the body of an infected individual with the number of ^{222}Rn atoms inhaled in their environment. According to Madas et al. [37] and Sender et al. [38], there are approximately 10^7 SARS-CoV-2 virions in the lungs of an infected person at any given time. If we consider the radon concentration in some departments in France, where it is relatively high (see Table 3), such as in Lozère, where it is 264 Bq/m^3, we can calculate the number of ^{222}Rn atoms. Using the formula N = ($T_{1/2}$ × A) / 0.693, where N represents the number of atoms, $T_{1/2}$ is ^{222}Rn physical half-life, and A is the radioactivity, we arrive at a value of 1.25 × 10^8 ^{222}Rn atoms. Since an average adult inhales approximately 23 m^3 of air per day [11], the total number of ^{222}Rn atoms inhaled would be 2.9 × 10^9, which is almost 300 times greater than the 10^7 SARS-CoV-2 virions. This suggests that the probability of a virion being hit by an alpha particle emitted during ^{222}Rn decay becomes significant. This interaction might provide an additional explanation for the lower COVID-19 mortality observed in regions with relatively high ^{222}Rn concentrations. In addition to directly impacting the virions, alpha particles could also destroy infected cells in the lungs and airways, further contributing to the overall effect of ^{222}Rn in reducing COVID-19 mortality. Numerous studies [37, 39] discuss the localization of aerosol and droplet deposits, which may carry virions, and the distribution of ^{222}Rn atoms in the respiratory tract, both attached and unattached. While alpha particles can damage healthy lung cells, the long-term consequences of this damage, such as the potential development of lung cancer, typically require decades to manifest and should be considered separately from the acute condition of COVID-19.

Finally, historical precedents for the use of ionizing radiation to treat infections have been reviewed [40]. More recently, molecularly targeted radiation, in the form of radioimmunotherapy, has been demonstrated to be effective in treating experimental fungal, bacterial, and viral infections [41]. Labeling antibodies that specifically bind to SARS-CoV-2 virions with alpha-emitting radionuclides, such as Astatine (^{211}At) or Lead (^{212}Pb), could be an effective way to deliver cytotoxic alpha particles to the surface of virions for their elimination.

Limitations of the study

Indeed, the limitations of ecological studies are well known (Canadian Nuclear Safety Commission-Basic epidemiology concepts) but it is just a misguided scientific attitude to reject them regarding a pandemic which is not under control. John Snow, who is considered as the father of epidemiology, observed that the area served by water from the Broad Street fountain in London matched with the cholera pandemic (august 1854) found a correlation which led to much less fatalities. It will be a mistake to ignore the Hill's Criteria as causation analysis of this work (Hill's criteria are cited since 1965 with an exponential number of publications since 2000 (Google Scholar)). Going through these criteria, we find the following estimation of concordance as follow:

Strength

There are several correlations, moderately weak but repetitive (<-0,5), at countries population level between the binary data - "deaths rates" of the COVID-19 - and the exposures to an environmental data "the indoor Radon in dwellings" which is an

extremely complex variable of geogenic and anthropogenic origins (Fig 1). The difficulty lies finding geographical areas with indoor Radon dwellings measurements matching administrative areas with daily recorded COVID-19 deaths rates. The analysis covers 10 countries having such property over 3 continents with populations close to half billion individuals during the first year of the pandemic. The repetitiveness shows a possible association.

Consistency

The fact that both the longitudinal observed values and the correlations values at different dates are repeatable highlights a possible association. All the data concerns the spread of the pandemic which started everywhere nearly at the same time in March 2020 (first reported cases) up to mid-December 2020 when massive vaccination started.

Specificity

The existence of a short-term incidence (within month) between the indoor Radon exposure and the deaths rates has no common link with the long term (tenths of years) lung cancerogenesis of it.

Temporality

Indoor Radon dwellings is a lifelong type of exposure which was already affecting the populations in the concerned areas when the pandemic started.

Biological Gradient

Both, Table 2 and the correlations show that an increase in the indoor Radon dwelling exposure show a monotonic negative relationship with deaths rates.

Plausibility and Coherence

The effect of radiations to enhance the immunitary system and killing virus and microbes is widely recognized in the literature.

Analogy

Thermal waters have been used for centuries to improve certain diseases, particularly those of genetic origin; X-rays empiric treatment of pneumonia with 30% of success before the discovery of the penicillin; Clinical tests for the treatment of the COVID-19 using X-Rays with spectacular fast recoveries in several countries.

Since the first work of Hill's in 1965, several authors have criticized Hill's criteria on the basis that several epidemiological studies gave contested conclusions, some of these are reported, following the work of Rothman [42] and summarized in the work of George Davey Smith [43].Regarding environmental epidemiology, where many parameters are not known and not measured, the use of randomized control trials (RCT) can not be used, and other approaches must be used as exposed in Pearce and al. [44]. One of his approaches, in our context, might be the triangulation of epidemiological evidence (as quoted in the Analogy, Plausibility and Coherence criteria). "Criteria for its use in causal inference in epidemiology have been proposed recently, and these specify that results from at least two (but ideally more) methods that have differing key sources of unrelated bias be compared. If evidence from such different epidemiologic approaches all point to the same conclusion, this strengthens confidence that is the correct causal conclusion, particularly when the key sources of bias of some of the approaches would predict that the findings would point in opposite directions" [44].

Data reliability

Except for France and Kerala where the data come from the governmental agencies, all other data come from the Johns Hopkins University (JHU). This university has established the Corona Resources Center (CRC) with the support of the Blomberg Philanthropies and the Stavros Niarcos Foundation (SNF) to collect and analyze the best available data on cases, deaths, tests, hospitalizations and vaccines to assist all stakeholders in the pandemic. In 2021, Research America presented CRC with the "Meeting the Moment for Public Health" award. Data is collected daily from every country in the world. Mortality results are provided in terms of the number of deaths per 100,000 population (country population). It is the latter ratio that we use since it is consistent with the radon exposure data that concern the whole population in each territory. It is clear that data from countries with high quality health services and statistics are as reliable as possible. This is especially true of the countries we have selected for this study. It is quite remarkable that the data for Kerala in India are also considered extremely reliable by other authors and have been used to reconsider the global statistics of deaths provided by India in general [45]. The CRC has reservations about the accuracy of some of the data provided by some countries.

Other environmental factors and possible bias

The relationship between the disease contracted and its fatal outcome is influenced by various biases, many of which are difficult to analyze, such as contamination, age and comorbidities. Contamination, roughly estimated by population density, has already been discussed in the matched analysis.

With regard to age, it is particularly relevant to examine the impact of radon exposure on the elderly. Research consistently shows that COVID-19 patients are more likely to suffer serious illness than younger individuals, often resulting in hospitalization. Therefore, examining mortality rates alone may be insufficient to attempt to estimate this bias. It may be more instructive to compare hospitalization rates rather than mortality rates. French daily data, which record both discharges home and deaths in hospital, can be used to analyse hospitalization rates.

We compared indoor radon data from different departements with the percentage of people hospitalized. Table 10 shows a similar trend for the hospitalization rates as for the mortality rates during the period studied, with a correlation coefficient close to -0.32. Notably in France, the correlation between the departements indoor radon exposure and the proportion of people aged 65 and over living there is 0.595. This indicates that a higher percentage of older adults are present in departments with higher indoor radon concentrations due to geographical and sociological contexts (corroborating results also observed in the USA).

Table 10. Spearman correlation results for the same dates as view previously

Spearman Correlation: Radon and Hospitalized Persons Average Rank					
**Date (dd/mm/yyyy)**	**11/06/2020**	**17/08/2020**	**01/09/2020**	**30/10/2020**	**12/12/2020**
**Spearman Correlation Coef.**	-0.313	-0.327	-0.333	-0.361	-0.228

Analyzing density presents more complexity than addressing age bias. The mean density within a department may not serve as a reliable indicator of contamination levels. However, it remains worth exploring these potential biases implications. Future investigations should incorporate comprehensive data on a range of potential confounders to strengthen the results. Although this study provides valuable information, it is important to recognize its limitations. Therefore, we recommend further research to explore this topic in greater depth.

Conclusion

Given the complexity of the various parameters that may influence the results of this research, along with its potentially disruptive nature, it is essential to exercise caution when drawing causal inferences about Radon exposure and its potential therapeutic effects on COVID-19 outcomes. Nevertheless, our study has shown a positive correlation between residential exposure to Radon and reduced morbidity from COVID-19 during the pre-vaccination period across three continents. This finding aligns with both historical and recent clinical observations indicating that low-dose ionizing radiation can reduce lung inflammation caused by infectious agents. However, the molecular mechanisms underlying this phenomenon require further investigation. Additionally, these results suggest an intriguing possibility for the future use of molecularly targeted radiation, such as radio-immunotherapy, in the treatment of COVID-19.

Supporting information

S1 Table. Analysis of Mortality rate and Radon Exposure in Relation to Population Density. This table provides a detailed breakdown of the statistical analysis, exploring the relationship between population density, radon exposure, and mortality rates across various regions in France using a matching pair differential.

S2 Table. Analysis of Mortality rate and Radon Exposure in Relation to Hospitalization Number in France. This table provides a detailed breakdown of the statistical analysis, exploring the relationship between population density, radon exposure, and the total hospitalization number across various regions in France using matching pair differential.

Data Availability

The datasets used in this study are publicly available on the following data servers:

- `https://www.data.gouv.fr/fr/datasets/donnees-hospitalieres-relatives-a-lepidemie-de-covid-19-en-france-1/`
- `https://www.irsn.fr/fr/connaissances/Environnement/expertises-radioactivite-naturelle/radon/Pages/4-Campagne-nationale-mesure-radon.aspx`
- `https://www.ecdc.europa.eu/`
- `http://europa.eu.int/`
- `https://github.com/nytimes/covid-19-data`
- `https://www.census.gov/data/tables/time-series/dec/density-data-text.htm`
- `https://www.insee.fr/fr/statistiques/189319`
- `http://publications.jrc.ec.europa.eu/repository/handle/JRC76737`
- `https://dashboard.kerala.gov.in/`
- `http://dashboard.kerala.gov.in/dailyreporting-view-public-districtwise.php`

Software Used

The following software was used in the study:

- `http://www.real-statistics.com`
- RStudio version 2023.12.1+402

Competing interest

The authors did not receive any funding to conduct this study.

Conflicts of interest

The authors certify that they have no affiliation with or involvement in any organization or entity with any financial or non-financial interest in the subject matter discussed in this manuscript.

Authors contributions

JFC designed the study based on his observations at the start of the pandemic and his knowledge of the nuclear environment. Together with GM, they produced the main body of this research. ED reviewed it, drawing on her expertise in Radiobiology. She contributed her understanding of the results and emphasized the biomedical perspectives they offer. SJ also reviewed the study, applying her expertise in Epidemiology and Statistical analysis to strengthen the findings.

References

1. Robert A. Coronavirus: un premier foyer identifié dans le département du Morbihan. Agence de Santé Bretagne. 2020;2.

2. Nabil M. Simultaneous measurement of Radon and Thoron released from building material used in Japan. Prog Nucl Sci Technol. 2011;1:404–407.

3. Uljić P. Internal exposure from building materials exhaling 222Rn and 220Rn as compared to external exposure due to their natural radioactivity content. Appl Radiat Isot. 2010;68:201–206.

4. Becker K. One century of radon therapy. Int J Low Radiat. 2004;1(3):225–250.

5. Franke A, Reiner L, Pratzel HG, Franke T, Resch KL. Long-term efficacy of radon spa therapy in rheumatoid arthritis—a randomized, sham-controlled study and follow-up. Rheumatology (Oxford) [Internet]. 2000;39(8):894–902. Available from: `http://dx.doi.org/10.1093/rheumatology/39.8.894`

6. Hess CB, Buchwald ZS, Stokes W, Switchenko JM, Nasti TH, Weinberg BD, et al. Low-dose whole-lung radiation for COVID-19 pneumonia: Planned day-7 interim analysis of an ongoing clinical trial [Internet]. 2020. Available from: `http://dx.doi.org/10.1101/2020.06.03.20116988`

7. Mortazavi SMJ, Shams SF, Mohammadi S, Mortazavi SAR, Sihver L. Low-Dose Radiation Therapy for COVID-19: a Systematic Review. Radiat. 2021;1:234–249.

8. Sadiq Aliyu A, Thermisi Rameli A. The world's high background natural radiation areas (HBNRAs) revisited: A broad overview of the dosimetric, epidemiological and radiobiological issues. Radiat Meas. 2015;73:51–59.

9. United Nations Scientific Committee on the Effect of the Atomic Radiations (UNSCEAR). Report of the Sixty-fifth Session. 2018;11–14.

10. Ajrouche R, Roudier C, Cléro E, Ielsch G, Gay D, Guillevic J, et al. Quantitative health impact of indoor radon in France. Radiat Environ Biophys [Internet]. 2018;57(3):205–214. Available from: http://dx.doi.org/10.1007/s00411-018-0741-x

11. EPA. A Citizen's Guide to Radon. 2016.

12. Schumann R, Editor R, editors. Geological Radon Potential of EPA Region 1 et seq., Open File Report 93-292-A et seq. - Prepared in Cooperation with the. U.S. Environmental Protection Agency; 1993.

13. Bossew P. The European radon mapping project. Radiat Prot Dosimetry. 2013;157(3):522–530.

14. Gambard JP, Mitton N, Pirard P. Atlas Radon, Notes Techniques SEGR/LEADS-2000/14. 2013.

15. Pantelić G, Čeliković I, Živanović M, Vukanac I, Nikolić JK, Cinelli G, et al. Qualitative overview of indoor radon surveys in Europe. J Environ Radioact [Internet]. 2019;204:163–174. Available from: http://dx.doi.org/10.1016/j.jenvrad.2019.04.010

16. Dubois GAG. An overview of radon survey in Europe, European Commission-JRC-Office for Official Publications of the European Communities. 2005.

17. Predrag U. Internal exposure from building material exhaling 222Rn and 220Rn as compared to external exposure due to their natural radioactivity content. Appl Radiat Isot. 2010;68:201–206.

18. Cinelli G, De Cort M. Joint Research Centre - European Atlas of Natural Radiation, Publication Office of the European Union. Tollefsen T, editor. Luxembourg; 2019.

19. Mtsariashvili L. Indoor Radon and features of its distribution in Tbilisi - The main city of Georgia. Proc of the 13th Int Multidisciplinary Scientific GeoConference: SGEM; Albena-Bulgaria. 2013;1:1235–1242.

20. Mortazavi SMJ, Ikuhima T, Mozdarani H, Sharafi AA, Ishi Y. La pré-irradiation de bas niveau des lymphocytes humains est-elle un phénomène absolument bénéfique? Un rapport sur le synergisme extra-ordinaire. Kowsar Med J. 2000;5(4):235–240.

21. Dong E, Du H, Gardner L. An interactive web-based dashboard to track COVID-19 in real time. Lancet Infect Dis [Internet]. 2020;20(5):533–534. Available from: http://dx.doi.org/10.1016/s1473-3099(20)30120-1

22. Bergsten JW, Holt NA, Lucas RM, Smith ML. The National Residential Radon Survey-Design Report. 1989.

23. Laurencelle L. De Kendall pour la correlation de variables ordinales simples ou catégorielles. Tutorials in Quantitative Methods for Psychology. 2009;5:51–58.

24. Zaiontz C. Real Statistics Using Excel. Real Statistics Resources. 2020.

25. Brazauskas R, Logan BR. Observational studies: Matching or regression? Biol Blood Marrow Transplant [Internet]. 2016;22(3):557–563. Available from: http://dx.doi.org/10.1016/j.bbmt.2015.12.005

26. Stuart EA. Matching methods for causal inference: A review and a look forward. Stat Sci [Internet]. 2010;25(1):1–21. Available from: http://dx.doi.org/10.1214/09-STS313

27. Rubin DB, Thomas N. Matching using estimated propensity scores: Relating theory to practice. Biometrics [Internet]. 1996;52(1):249–264. Available from: http://dx.doi.org/10.2307/2533160

28. Kabat G. Germany and Austria, Visits to Radon Health Spas are Covered By Health Insurance. Sci Technol Forbes Media LLC. 2019;2002868355–9.

29. Améon R. Le radon dans les stations thermales: une source d'exposition aux rayonnements ionisants. Radioprotection [Internet]. 2003 [cited 2024 Sep 18];38:201–215. Available from: https://www.dissident-media.org/infonucleaire/Ameon.pdf

30. Kirkby C, Mackenzie M. Is low dose radiation therapy a potential treatment for COVID-19 pneumonia? Radiother Oncol [Internet]. 2020;147:221. Available from: http://dx.doi.org/10.1016/j.radonc.2020.04.004

31. Venkatesulu BP, Lester S, Hsieh C-E, Verma V, Sharon E, Ahmed M, et al. Low-dose radiation therapy for COVID-19: Promises and pitfalls. JNCI Cancer Spectr [Internet]. 2021;5(1):kaa103. Available from: http://dx.doi.org/10.1093/jncics/pkaa103

32. Salomaa S, Cardis E, Bouffler SD, Atkinson MJ, Hamada N. Low dose radiation therapy for COVID-19 pneumonia: is there any supportive evidence? Int J Radiat Biol [Internet]. 2020;96(10):1224–1227. Available from: http://dx.doi.org/10.1080/09553002.2020.1762020

33. Schaue D, McBride WH. Flying by the seat of our pants: Is low dose radiation therapy for COVID-19 an option? Int J Radiat Biol [Internet]. 2020;96(10):1219–1223. Available from: http://dx.doi.org/10.1080/09553002.2020.1767314

34. Cosset J-M, Deutsch É, Bazire L, Mazeron J-J, Chargari C. Irradiation pulmonaire à faible dose pour l'orage de cytokines du COVID-19: pourquoi pas? Cancer Radiother [Internet]. 2020;24(3):179–181. Available from: http://dx.doi.org/10.1016/j.canrad.2020.04.003

35. Clayton B. Low-Dose Whole-Lung Radiation for COVID-19, Pneumonia. medRxiv preprint. 2020.

36. Bahsoun A, Fakih Y, Zareef R, Bitar F, Arabi M. Corticosteroids in COVID-19: Pros and cons. Front Med (Lausanne) [Internet]. 2023;10:1202504. Available from: http://dx.doi.org/10.3389/fmed.2023.1202504

37. Madas BG, Füri P, Farkas Á, Nagy A, Czitrovszky A, Balásházy I, et al. Deposition distribution of the new coronavirus (SARS-CoV-2) in the human airways upon exposure to cough-generated droplets and aerosol particles. Sci Rep [Internet]. 2020;10(1):22430. Available from: http://dx.doi.org/10.1038/s41598-020-79985-6

38. Sender R, Bar-On YM, Gleizer S, Bernshtein B, Flamholz A, Phillips R, et al. The total number and mass of SARS-CoV-2 virions. Proc Natl Acad Sci U S A [Internet]. 2021;118(25). Available from: http://dx.doi.org/10.1073/pnas.2024815118

39. Hofmann W, Bolt L, Sturm R, Fleming JS, Conway JH. Simulation of three-dimensional particle deposition patterns in human lungs and comparison with experimental SPECT data. Aerosol Sci Technol [Internet]. 2005;39(8):771–781. Available from: http://dx.doi.org/10.1080/02786820500237158

40. van Dijk B, Lemans JVC, Hoogendoorn RM, Dadachova E, de Klerk JMH, Vogely HC, et al. Treating infections with ionizing radiation: a historical perspective and emerging techniques. Antimicrob Resist Infect Control [Internet]. 2020;9(1). Available from: http://dx.doi.org/10.1186/s13756-020-00775-w

41. Carvalho JLC, Dadachova E. Radioimmunotherapy for the treatment of infectious diseases: a comprehensive update. Expert Rev Anti Infect Ther [Internet]. 2023;21(4):365–374. Available from: http://dx.doi.org/10.1080/14787210.2023.2184345

42. McGill.ca. Rothman S. Modern Epidemiology [Internet]. [cited 2024 Sep 18]. Available from: https://www.medicine.mcgill.ca/epidemiology/hanley/bios601/Rothman86ModernEpidemiology/Rothman86-00-front-material.pdf

43. Smith GD. Post-Modern Epidemiology: When Methods Meet Matter. Am J Epidemiol. 2019;188(8):1410–1419.

44. Pearce N, Vandenbroucke JP, Lawlor DA. Causal inference in environmental epidemiology: Old and new approaches. Epidemiology [Internet]. 2019;30(3):311–316. Available from: http://dx.doi.org/10.1097/EDE.0000000000000987

45. Guilmoto CZ. An alternative estimation of the death toll of the COVID-19 pandemic in India. PLoS One [Internet]. 2022;17(2):e0263187. Available from: http://dx.doi.org/10.1371/journal.pone.0263187

Fig1 Click here to access/download;Figure;Fig1.tif

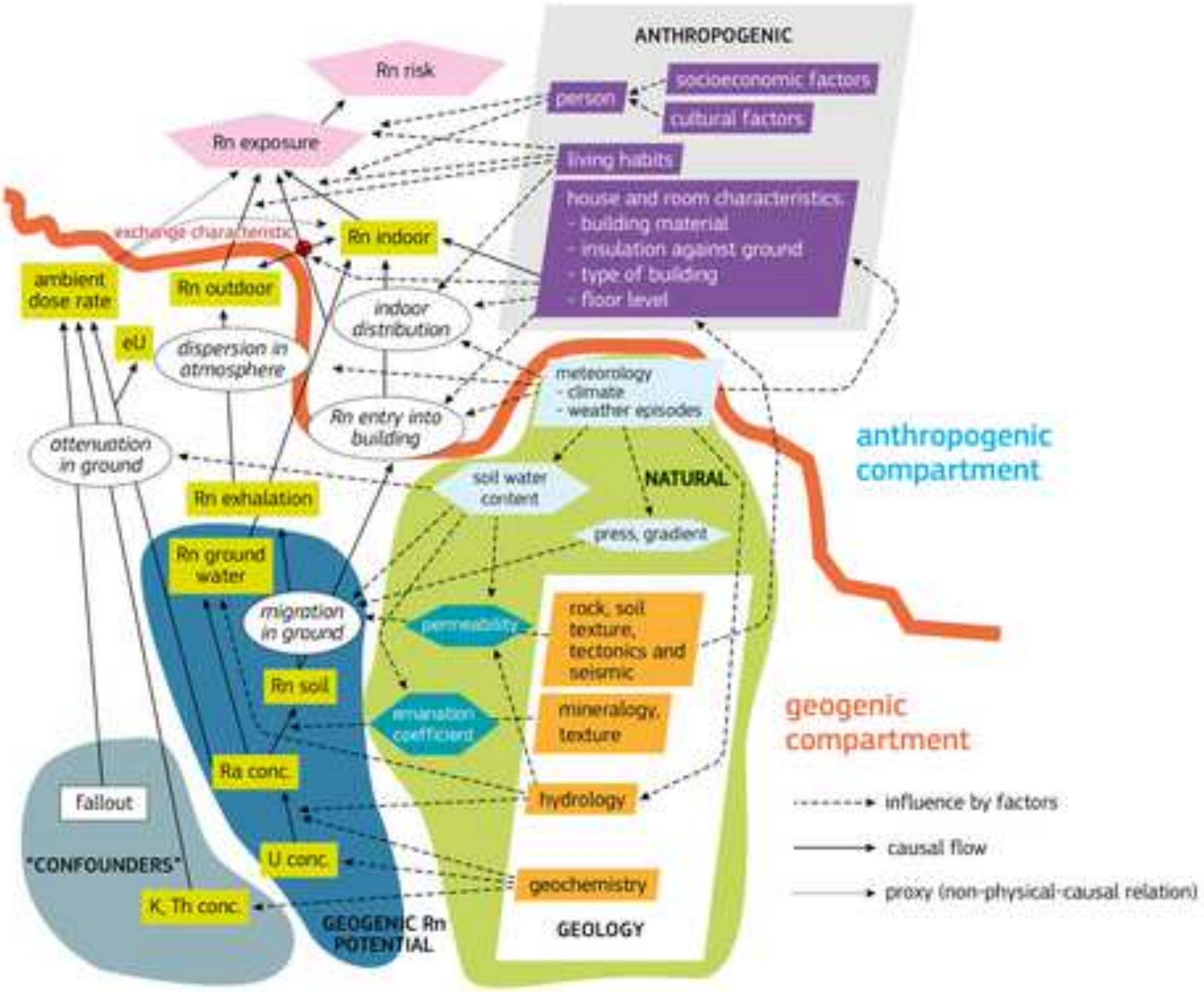

Fig2

Click here to access/download;Figure;Fig2.tif

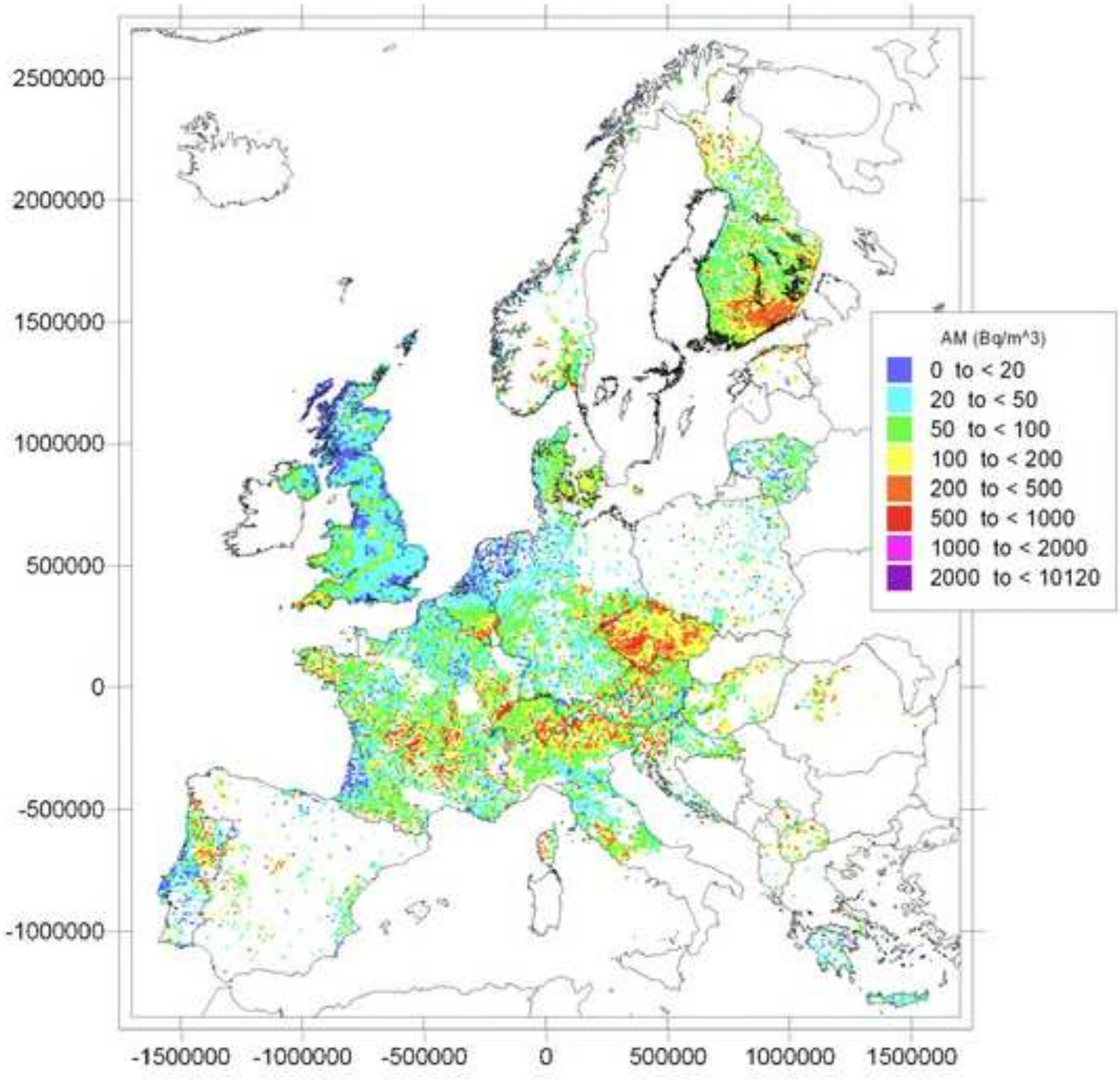

Fig3

Click here to access/download;Figure;Fig3.tif

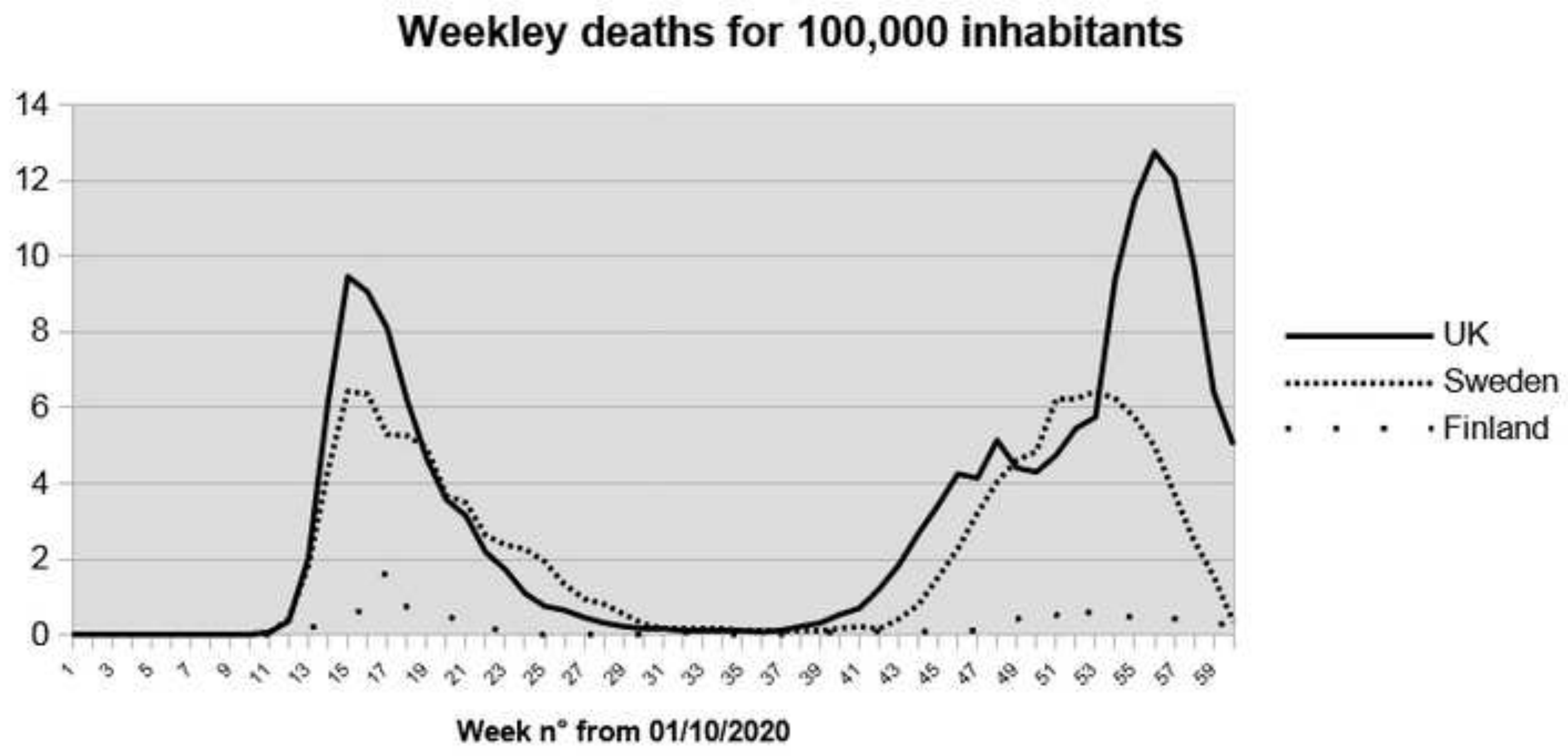

Fig4

Click here to access/download;Figure;Fig4.tif

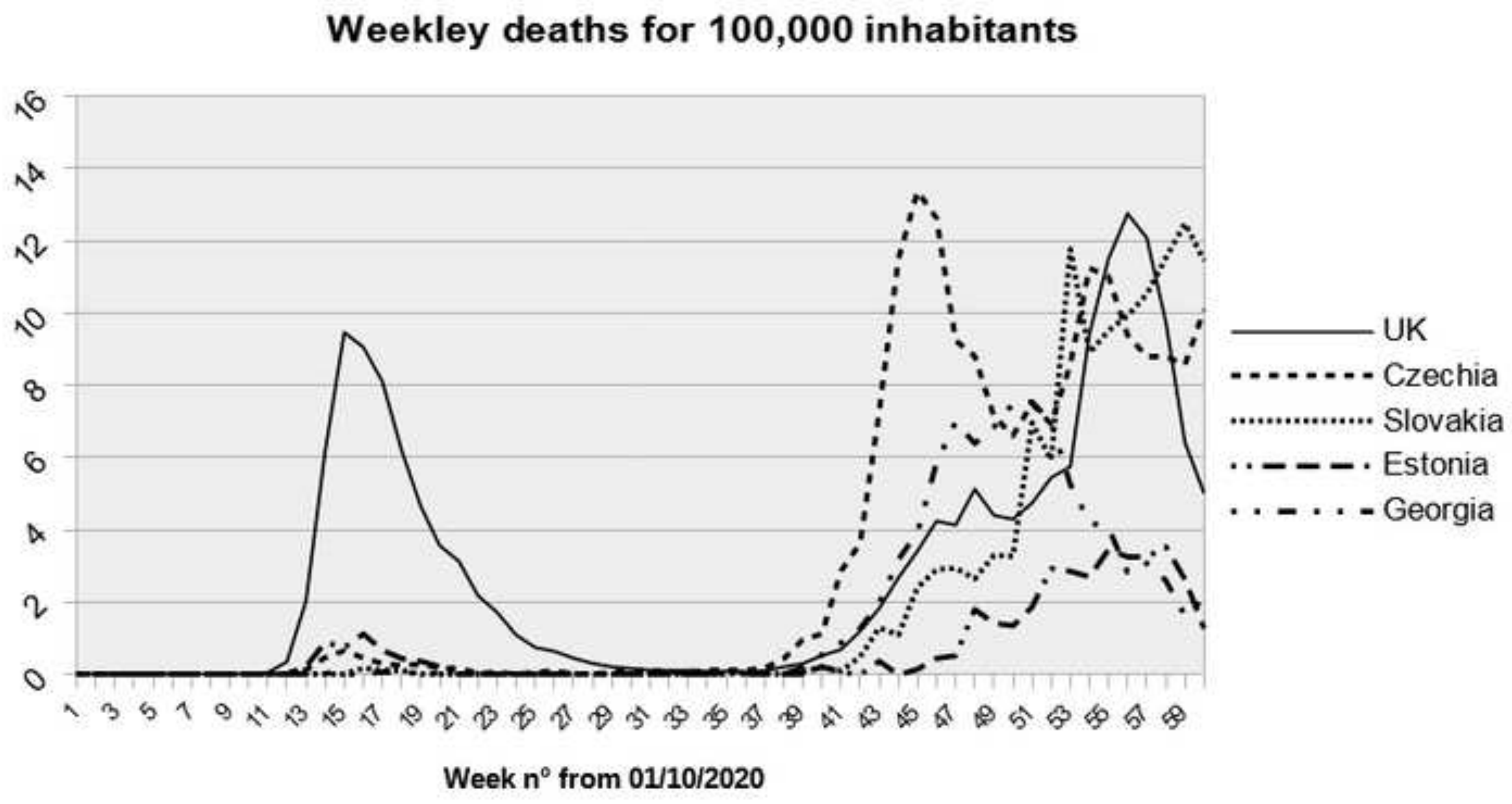

Fig5

Click here to access/download;Figure;Fig5.tif

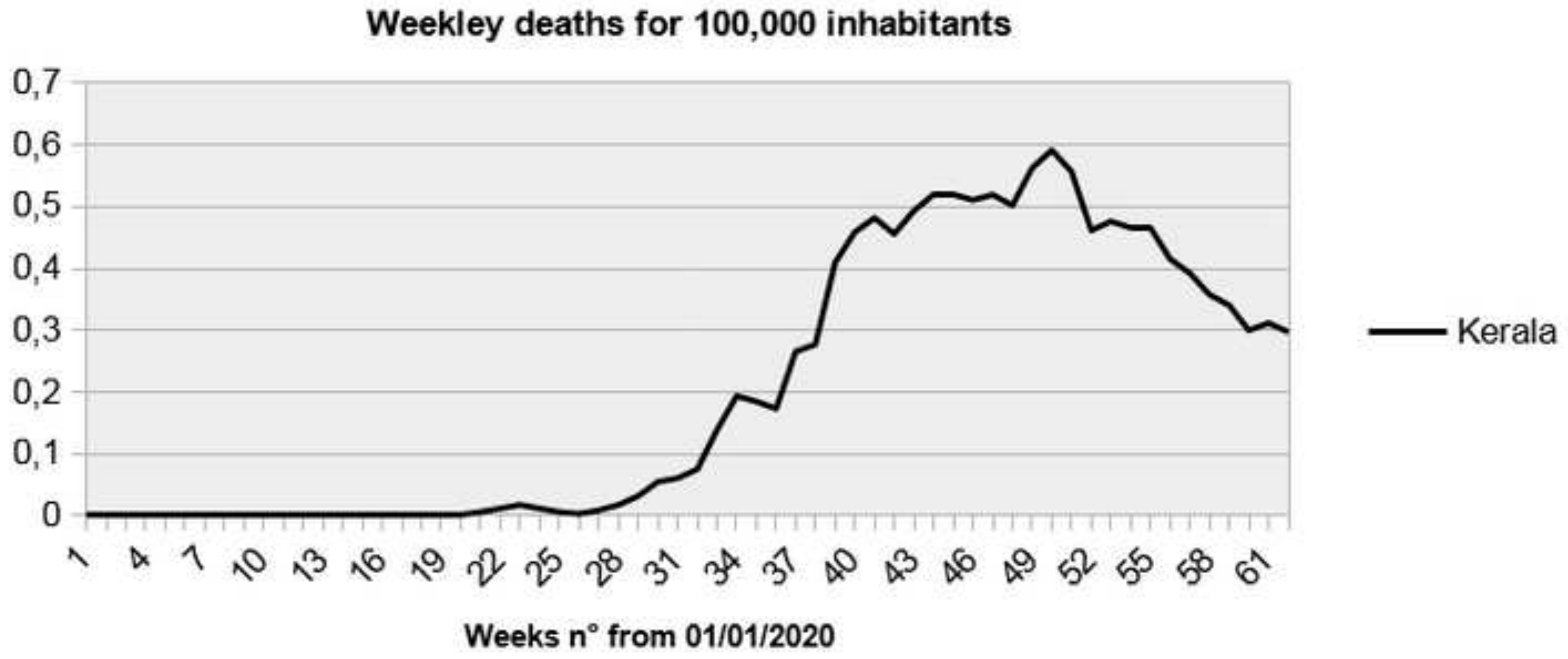

Fig6

Click here to access/download;Figure;Fig6.tif

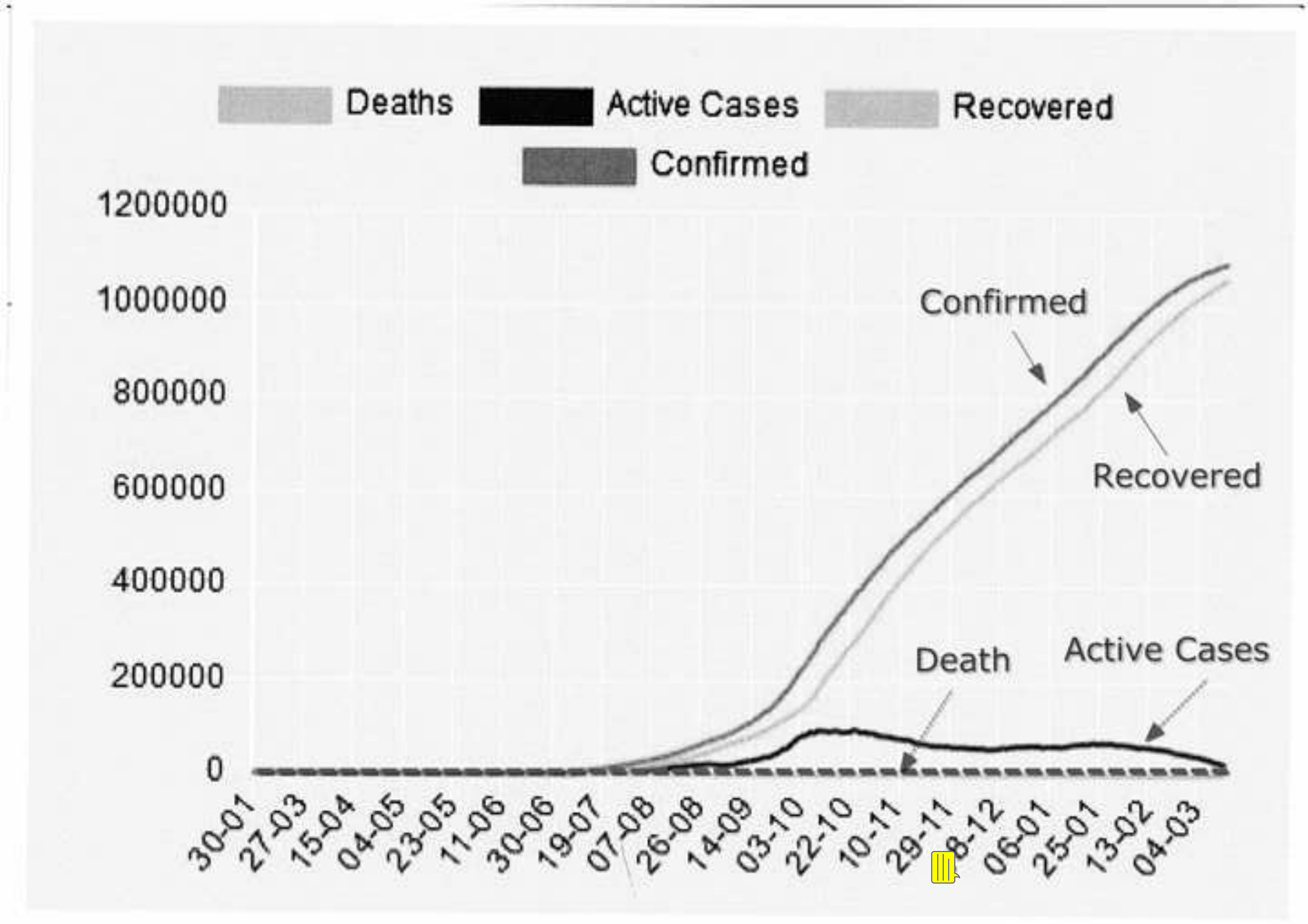

Fig7

Click here to access/download;Figure;Fig7.tif

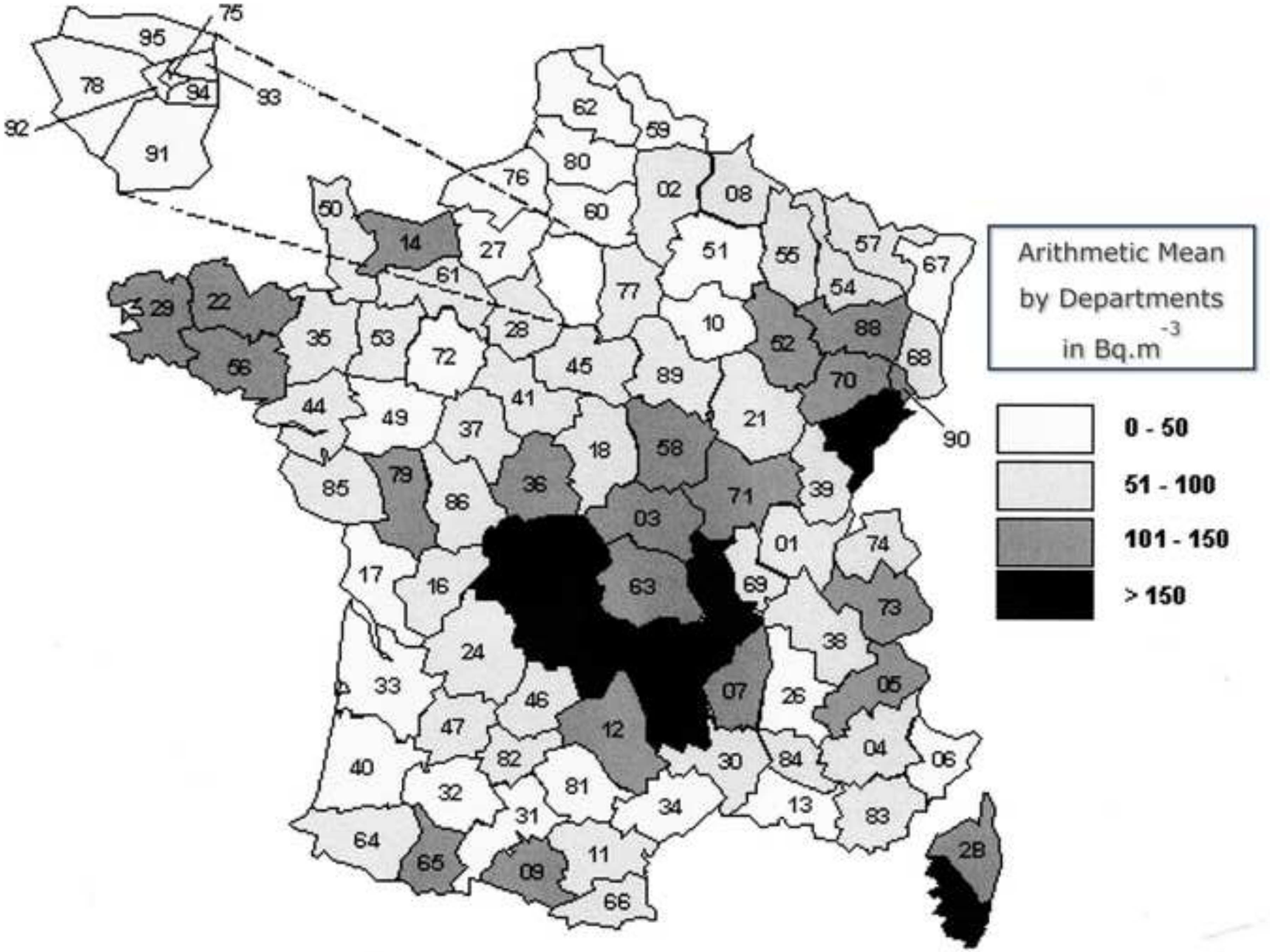

Click here to access/download;Figure;Fig8.tif

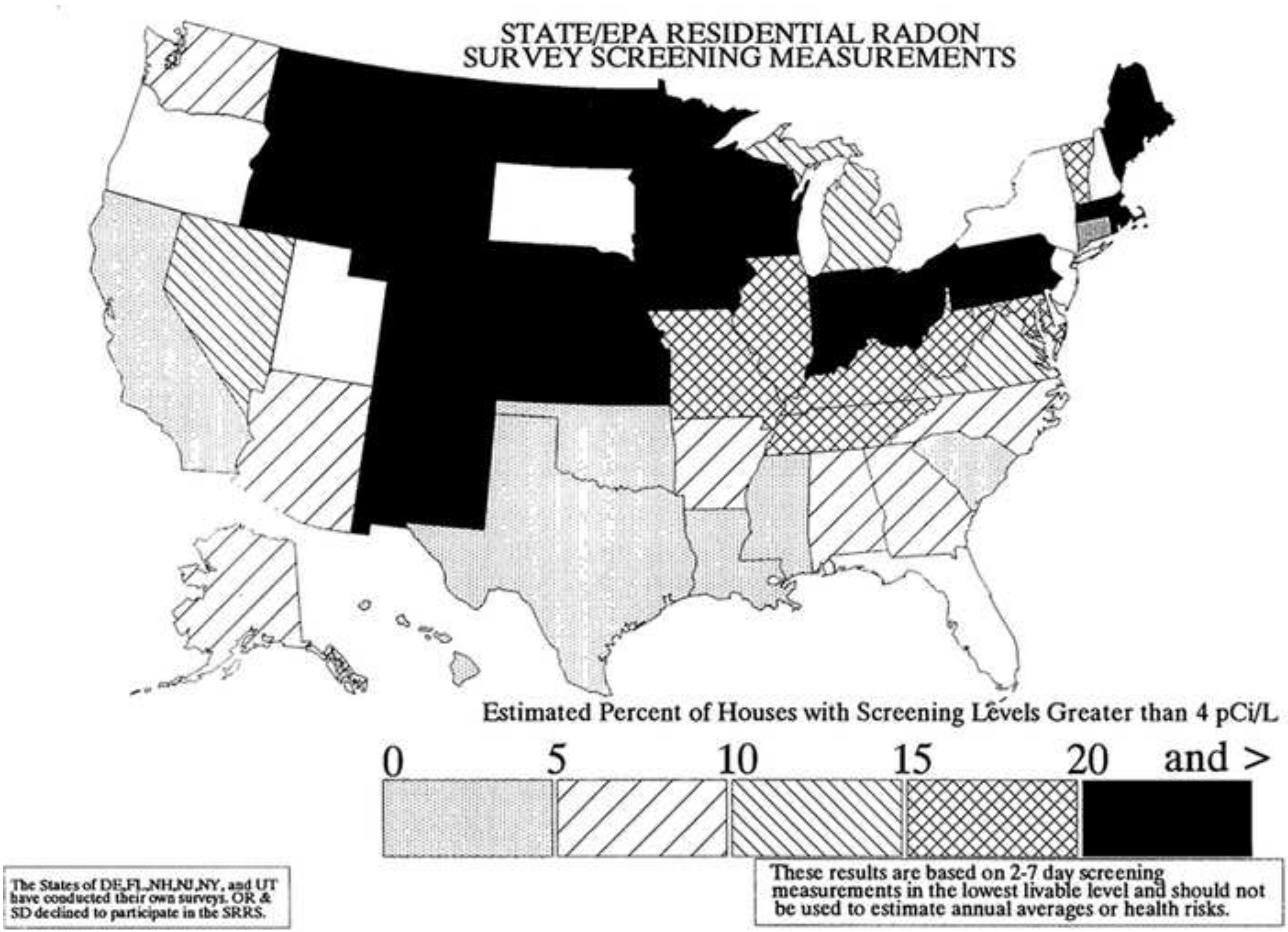

SI1

Click here to access/download
Supporting Information
Matching_PLOS_S1.pdf

Click here to access/download
Supporting Information
Matching_PLOS_S2.pdf
