# Supplementary material for: Radon exposure and COVID-19 mortality in pre-vaccination period: What links might exist?
Source: PLoS One. 2025 Dec 5;20(12):e0337320. doi: 10.1371/journal.pone.0337320 (PMC12680143; doi:10.1371/journal.pone.0337320)
Supplement: Attachment — Submitted filename: Rebuttal letter to reviewer_PLOSONE.docx [file pone.0337320.s004.docx]

**Response to Reviewers – Manuscript PONE-D-24-57007**
**Title:** ***Radon exposure and COVID-19 mortality in pre-vaccination period: what links might exist?***

Dear Academic Editor and Reviewers,

We sincerely thank you for your time, your positive evaluations, and the constructive comments provided during the review of our manuscript. We are pleased that both reviewers found the manuscript scientifically sound, well-written, and suitable for publication pending minor revisions.

Please find below our detailed point-by-point responses. All modifications have been incorporated into the revised manuscript and tracked accordingly in the submitted version with changes.

**General Reviewer Feedback**

**Reviewer 1**:

*“This unique research has the potential to pave the way for further studies... Based on its merit and well-executed design, I recommend acceptance of this manuscript without any modifications.”*

**Response**:
We thank Reviewer 1 for the positive feedback and support for our manuscript. No specific changes were requested.

**Reviewer 2**:

*“The manuscript is well written in clear English. However, there are minor grammatical suggestions highlighted in the reviewed manuscript. Also, the introduction section is very lengthy. Some sections may be shifted to the discussion section, like data and discussion of Kerala.”*

**Response:
We appreciate Reviewer 2’s helpful suggestions. In response:**

1. **Grammar:** We have carefully addressed all grammatical suggestions as indicated in the reviewer’s annotated file. (Line 85)
2. **Introduction length:** We agree the introduction was too long. We have shortened it by relocating the Kerala-specific background and interpretation to the Discussion section (lines 236–263) where it fits better with our results. Additionally, some parts related to the statistical study using French data have been moved from the Materials and Methods to the Discussion (lines 265–271). Figure numbering has been updated accordingly.
3. **Clarification in the abstract:**
   - *Original:* “The findings revealed a consistent negative correlation between higher radon concentrations and lower COVID-19 mortality rates.”
   - *Revised:* “The findings revealed a consistent negative correlation between radon concentrations and COVID-19 mortality rates, indicating that higher radon concentrations were associated with lower mortality rates.”

**Editorial and Journal Requirements**

1. **Style & Formatting:**
   We have reviewed the PLOS ONE formatting guidelines and updated the manuscript and files to meet the style requirements, including proper file naming.
2. **Figures 2 & 8 — Map image copyright:**

**Figure 2 (European Atlas of Natural Radiation):**
We confirm that Figure 2 is adapted from the European Atlas of Natural Radiation by the European Commission’s Joint Research Centre (JRC). According to the European Commission Decision of 12 December 2011 on the reuse of Commission documents, this material is available under the Creative Commons Attribution 4.0 International (CC BY 4.0) licence unless otherwise indicated.

**Figure 1. Description of the phenomenon involved in the risk evaluation of indoor radon.** We received permission from the author, Peter Bossew, to reuse this figure, and we have attached the permission document accordingly.

**Figure 8 (USGS–EPA Open-File Report 93-292 previously figure 8. After revisions, it is figure 6):**
We confirm that Figure 8 is reproduced from the USGS–EPA Open-File Report 93-292, which is an official publication by U.S. federal agencies. Under U.S. law (17 U.S.C. § 105), works created by the U.S. government are in the public domain and are not subject to copyright restrictions.
To comply with PLOS ONE’s policy, we have:

- Verified that this material is in the public domain.
- Updated the figure caption to include a clear public domain notice and correct source attribution.

We hope this clarifies the licensing status of both figures and we remain available should the editorial team require any further information.

3. **References:**
   We have systematically checked all 45 references in five batches. None are retracted or under expression of concern. All DOIs are valid and the articles remain indexed on PubMed, PMC, and publisher sites.
4. **Minor reference updates:**
   We have updated a few references for completeness and corrected minor formatting issues. These changes are tracked in the revised manuscript:
   - Page 5 – source: ECDC, 2020
   - Line 157 – INSEE, 2020
   - Figure 6 – X and Y variable are now labelled

We hope that our responses and revisions meet your expectations and would like to thank you again for your time and consideration.

Kind regards,
**Stephanie Jonathan, M.D.**
(On behalf of all co-authors)
